# Revision of the afrotropical species of *Zaprionus* (Diptera, Drosophilidae), with descriptions of two new species and notes on internal reproductive structures and immature stages

**DOI:** 10.3897/zookeys.51.380

**Published:** 2010-07-23

**Authors:** Amir Yassin, Jean R. David

**Affiliations:** 1 Laboratoire Evolution, Génomes et Spéciation (LEGS), Centre National de la Recherche Scientifi que (CNRS), av. de la Terrasse, 91198 Gif-sur-Yvette Cedex; Université Paris-Sud XI, 91400 Orsay; France; 2 Sackler Institute for Comparative Genomics, Department of Invertebrate Systematics, American Museum of Natural History (AMNH), New York, USA; 3 Département Systématique et Evolution, Muséum National d’Histoire Naturelle (MNHN), Paris, France

**Keywords:** classification, reproductive system, immature stages, taxonomy, cryptic species, Tropical Africa

## Abstract

A new classification of the subgenus Zaprionus is proposed in light of recent phylogenetic findings. The boundaries of the armatus and inermis species groups are redefined. The vittiger subgroup is upgraded to the level of a species group. The tuberculatus subgroup is transferred from the armatus to the inermis group. A new monotypic group, neglectus, is erected. Full morphological descriptions of four species belonging to the vittiger group are given: Zaprionus lachaisei **sp. n.** from Tanzania and Zaprionus santomensis **sp. n.** from São Tomé and Principé, and two cryptic species of the indianus complex, Zaprionus africanus Yassin & David and Zaprionus gabonicus Yassin & David. Three nominal species are synonymised: Zaprionus beninensis Chassagnard & Tsacas, **syn. n.** with Zaprionus koroleu Burla, Zaprionus simplex Chassagnard & McEvey, **syn. n.** with Zaprionus neglectus Collart, and Zaprionus megalorchis Chassagnard & Tsacas, **syn. n.** with Zaprionus ornatus Séguy. Half of the 46 species of the subgenus are available as laboratory strains and this has allowed full descriptions of the internal structure of their reproductive systems and their immature stages.

## Introduction

**Figure 1:**
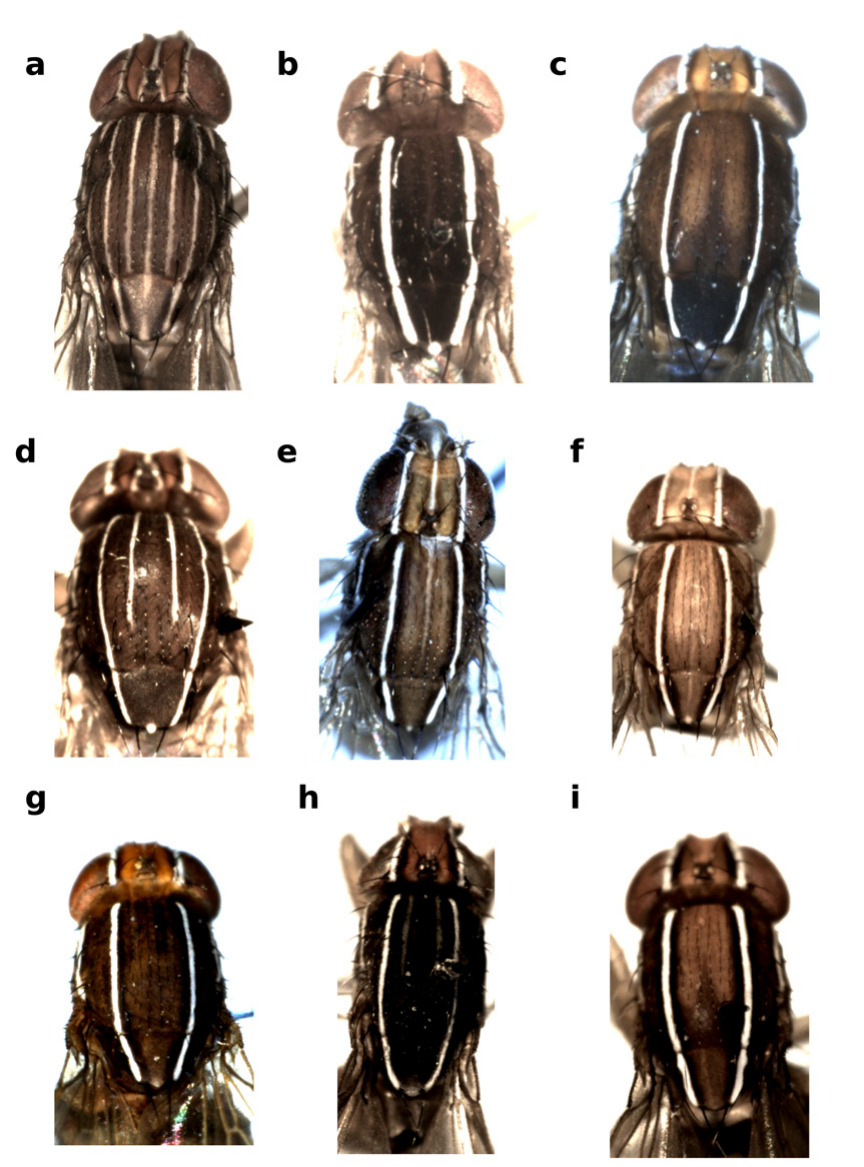
Frons and mesonotum of Zaprionus (Anaprionus) bogoriensis Mainx, 1954 **a**, Zaprionus (Zaprionus) ghesquierei Collart, 1937a **b**, Zaprionus (Zaprionus) litos Chassagnard & McEvey, 1992 **c**, Zaprionus (Zaprionus) sexstriatus Chassagnard, 1996 **d**, Zaprionus (Zaprionus) cercus Chassagnard & McEvey, 1992 **e**, Zaprionus (Zaprionus) kolodkinae Chassagnard & Tsacas, 1987 **f**, Zaprionus (Zaprionus) verruca Chassagnard & McEvey, 1992 **g**, Zaprionus (Zaprionus) multivittiger Chassagnard, 1996 **h**, and Zaprionus (Zaprionus) davidi Chassagnard & Tsacas, 1993 **i**.

The drosophilid genus Zaprionus Coquillett, 1902 is characterized by the presence of longitudinal white stripes on the frons and the mesonotum (Fig. 1). It is a Paleotropical genus whose species are classified under two subgenera: Zaprionus sensu stricto in the Afrotropical region (48 species), and Anaprionus in the Oriental and Australasian regions (11 species) (Okada and Carson 1983; Markow and O’Grady 2006; Brake and Bächli 2008). The two subgenera are distinguished on the basis of the number of their mesonotal stripes, being even in Zaprionus s.s. and odd in Anaprionus. Flies of the subgenus Zaprionus form an important component of the Afrotropical drosophilid fauna, in terms of number of species, relative abundance and large body size (Tsacas et al. 1981; Yassin and David in press). Chassagnard and Tsacas (1993) classified those species under two groups: the armatus group with ornamented forefemora, and the inermis group with unornamented forefemora. Recent phylogenetic revisions using molecular and morphological characters have shown Zaprionus s.s. species to be monophyletic, but both species groups to be polyphyletic (Yassin et al. 2008a, 2010, in press).

In this paper, we propose a new classification based on recent phylogenetic findings, describe two new species, and provide a taxonomic key to all African Zaprionus species. In the early 1990s, several taxonomic keys were published for African Zaprionus (Tsacas and Chassagnard 1990; Chassagnard and McEvey 1992; Chassagnard and Tsacas 1993), but these usually treated some species subgroups or geographical localities and covered only 76% of the then known species. Since 1993, eight species were described including the two new ones described here. Twenty three species were available as laboratory strains, and this allowed us to also provide descriptions of internal reproductive system and premature morphology.

## Materials and methods

### Specimens examined

Examined specimens were museum-preserved material or laboratory strains. Laboratory strains in the Laboratoire Evolution, Génomes et Spéciation (LEGS) belonged to 23 species (Table 1), and they were used in describing internal structures of the male and female reproductive systems and immature stages. As shown in Table 1, a congeneric Oriental species, Zaprionus (Anaprionus) bogoriensis Mainx, was added to the analysis.

**Table 1:** List of laboratory strains used in studying internal reproductive structures and immature stages.

Species Founder	females collection data
Subgenus Anaprionus	
Zaprionus bogoriensis Mainx	India: Bangalore; 2004, J. R. David
Subgenus Zaprionus s.s.	
Zaprionus africanus Yassin & David	Uganda: Kibale (1100 m); vii.2003, D. Lachaise
Zaprionus burlai Yassin	Tanzania: East Usambara Mt, Amani (900 m); 25-ix-2002, D. Lachaise
Zaprionus camerounensis Chassagnard & Tsacas	Tanzania: East Usambara Mt, Amani (900 m); 25-ix-2002, D. Lachaise
Zaprionus capensis Chassagnard & Tsacas	South Africa: Cape Town; ii.1984, J. R. David
Zaprionus cercus Chassagnard & McEvey	Madagascar: Maroantsetra; 18-26.x.1987, S. F. McEvey, J. R. David & S. Aulard
Zaprionus davidi Chassagnard & Tsacas	Congo: Brazzaville; iii.2006, J. Vouidibio
Zaprionus gabonicus Yassin & David	Gabon: Ogoué-Ivindo, Makoukou (500 m); i.2004, F. Mavoungou
Zaprionus ghesquierei Collart	Congo: Brazzaville; iii.2006, J. Vouidibio
Zaprionus indianus Gupta	Brazil: Rio de Janeiro, Tijuca; 2001, J. R. David
Zaprionus inermis Collart	Uganda: Kibale (1100 m); vii.2003, D. Lachaise
Zaprionus kolodkinae Chassagnard & Tsacas	Madagascar: Antananarivo, Tsimbazaza (1200 m); ii.2008, A. Yassin & J. R. David
Zaprionus lachaisei sp. n.	Tanzania: East Usambara Mt, Amani (900 m); 25-ix-2002, D. Lachaise
Zaprionus mascariensis Tsacas & David	La Reunion (France): 2004, P. Capy
Zaprionus neglectus Collart	Madagascar: Andasibe; ii.2008, A. Yassin & J. R. David
Zaprionus ornatus Séguy	Congo: Brazzaville; iii.2006, J. Vouidibiou
Zaprionus proximus Collart	Kenya: S. Dupas
Zaprionus santomensis sp. n.	Sao Tomé & Príncipe: Pico de São Tomé Park (1500 m); iii.2001, D. Lachaise
Zaprionus sepsoides Duda	Congo: Brazzaville; iii.2006, J. Vouidibiou
Zaprionus taronus Chassagnard & Tsacas	Kenya: S. Dupas
Zaprionus tsacasi Yassin	Sao Tomé & Príncipe: Pico de São Tomé Park (1500 m); iii.2001, D. Lachaise
Zaprionus tuberculatus Malloch	Congo: Brazzaville; iii.2006, J. Vouidibiou
Zaprionus verruca Chassagnard & McEvey	Madagascar: Antananarivo, Tsimbazaza (1200 m); ii.2008, A. Yassin & J. R. David
Zaprionus vittiger Coquillett	South Africa: Cape Province, Stellarbush; xii.2006, M. Debiais-Thibaud

### Morphological description

Formal morphological description of the new species followed standard Drosophila terminology and index formulae as in McEvey (1990). Specimens were deposited in Laboratoire Evolution, Génomes et Spéciation, Gif-sur-Yvette, France (LEGS) as living cultures, frozen and alcohol-preserved material and microscopic preparations, and in Muséum National d’Histoire Naturelle, Paris, France (MNHN) as pinned material.

Morphological structures are abbreviated as:fwfront width;flfront length;hwhead width;omaximum diameter of the eye;jwidth of gena in line with o;chmaximum width of gena;or1proclinate orbital seta;or2anterior reclinate orbital seta;or3posterior reclinate orbital seta;ococellar seta;pocpost-ocellar seta;ivinner vertical seta;ovouter vertical seta;acsacrostichal setulae;adcanterior dorsocentral;pdcposterior dorsocentral;pscprescutullar seta;bscbasal scutellar seta;ascapical scutellar seta;F1forefemur;WLwing length;Wlwing width;TLthorax length;WVwidth of white vittae at adc;BVwidth of black vittae surrounding WV at adc;Anumber of abdominal bristles summed over successive sternites. Measurements on immature stages were taken from uncrowded cultures grown under the same conditions (at 21°C). Measurements are abbreviated as:ELegg length;Elegg width;PFlength of egg posterior filament;PLpuparium length;Plpuparium width; H (horn index)the ratio of the length of the anterior spiracles to the total length of the puparium × 100.

### Anatomy of the internal reproductive system

Mature, about 10 days old adults were dissected in a Drosophila Ringer solution. For the male reproductive system (see drawings in Lachaise 1972; Araripe et al. 2004), testes were uncoiled before a linear measurement could be done. This operation was facilitated by allowing the Ringer solution to evaporate a little so that the testis loses its rigidity. Linear measurements were done with a stereomicroscope equipped with a micrometer. Six lengths were measured:TSTtestis;SVseminal vesicle;VDvas deferens;PARparagonia (accessory gland);ECejaculatory canal;EBejaculatory bulb; and CAEcaecum. PAR and EB are glandular structures and their measurements are variable according to the reproductive status of the dissected male. They do not provide thus reliable taxonomic information. For the female (cf. Lachaise 1972), the lengths of two organs were measured after dissection: SR = seminal receptacle and SP = spermatheca length. The SR also makes irregular coils at the junction between the oviduct and uterus, and was uncoiled with tiny needles before measurement. As with immature stages, two or three individuals from almost each species were measured and the results were very similar. Multiple measurements were not taken for all species, but slight differences were only found within those for which multiple measurements were taken.

### A key to African Zaprionus

**Table:** 

1	F1 without a row of spines (Fig. 2a,b)	2
–	F1 with a row of spines (Fig. 2c–f)	19
2(1)	F1 with a protruding tubercule bearing a bristle (Fig. 2b)	3
–	F1 without a protruding tubercule (Fig. 2a)	7
3(2)	Frons without a median white stripe; ♂A = 46–57; aedeagus subterminally concave (Fig. 3a); spermatheca smooth (Fig. 3c)	Zaprionus mascariensis [Madagascar; Mauritius; Mayotte (France) (**loc. n.**); La Réunion (France)]
–	Frons with a median white stripe; ♂A = 22–37; aedeagus subterminally convex (Fig. 3e,i); spermatheca rough (Fig. 3g,k)	4
4(3)	TST = 1–2 mm; spermatheca very papillate (Fig. 3g); posterior egg filament spatulate (Fig. 3h)	5
–	TST = 3–5 mm; spermatheca somewhat papillate (Fig. 3k); posterior egg filament not spatulate (Fig. 3l)	6
5(4)	♂WV = 1.5–1.8 μm; TST = 2.0 mm	Zaprionus sepsoides [Benin; Cameroon; Côte d’Ivoire; Gabon; Congo; Madagascar; Malawi; South Africa; Uganda]
–	♂WV = 1.9–2.5 μm; TST = 1.2 mm	Zaprionus tsacasi [São Tomé and Principé]
6(3)	TST = 3.2 mm	Zaprionus tuberculatus [Cameroon; Canary Islands (Spain); Cabo Verde; Central African Republic; Chad; Congo; Côte d’Ivoire; Cyprus; Democratic Republic of Congo; Egypt; Gabon; Greece; Kenya; Israel; Madagascar; Malawi; Malta; Mauritius; Mayotte (France) (loc. n.); Mozambique; Niger; Nigeria; La Réunion (France); Zambia; Seychelles; South Africa; St. Helena; Tanzania; Uganda; Zimbabwe]
–	TST = 4.4 mm	Zaprionus burlai [Tanzania]
7(2)	Frons without a median stripe	8
–	Frons with a median stripe	14
8(7)	Scutum velvety black, especially posteriorly; scutellum with a white spot at tip (Fig. 1b)	Zaprionus ghesquierei [Benin; Cameroon; Congo; Côte d’Ivoire; Democratic Republic of Congo; Gabon; Kenya; Madagascar; Malawi; Niger; Nigeria; São Tomé and Principé; Swaziland; Tanzania; Turkey; Uganda; Hawaii Islands (United States of America); Zimbabwe]
–	Scutum and scutellum not as above	9
9(8)	Scutellum entirely and scutum posteromedially black (Fig. 1c)	Zaprionus litos [Madagascar]
–	Scutellum and scutum not as above	10
10(9)	Wing darkened anteriorly	11
–	Wing uniformally hyaline	12
11(10)	Thorax and abdomen entirely dark brown (Fig. 4a)	Zaprionus momorticus [Côte d’Ivoire; Democratic Republic of Congo]
–	Thorax and abdomen yellow (Fig. 4b)	Zaprionus badyi [Côte d’Ivoire]
12(10)	♂ basitarsus without a hairy brush (Fig. 5a)	Zaprionus neglectus [Côte d’Ivoire; Democratic Republic of Congo; Madagascar]
–	♂ basitarsus with a hairy brush (Fig. 5b-d)	13
13(12)	Thorax yellow; the last 3 abdominal segments shining dark brown (Fig. 4c)	Zaprionus niabu [Côte d’Ivoire]
–	Thorax reddish yellow; abdomen shining yellow (Fig. 4d)	Zaprionus arduus [Côte d’Ivoire; Democratic Republic of Congo]
14(7)	Scutum with 6 longitudinal white stripes (Fig. 1d)	15
–	Scutum with 4 longitudinal white stripes (Fig. 1e)	16
15(14)	Aedeagal flap smooth and pointed basally (Fig. 6a)	Zaprionus sexvittatus [Democratic Republic of Congo; Kenya]
–	Aedeagal flap finely serrated and truncated basally (Fig. 6b)	Zaprionus sexstriatus [South Africa]
16(14)	Cercus with elongate, ventromedial expansion (Fig. 7a,b)	17
–	Cercus without ventromedial expansion (Figs 7c,d)	18
17(16)	Thorax with a faint median white stripe (Fig. 1e); ♂WL:TL = 2.02–2.15; abdomen with dark spots at the base of tergal bristles; cercal prominence long and basomedially setulate (Fig. 7b)	Zaprionus cercus [Madagascar]
–	Thorax without a faint median white stripe; ♂WL:TL = 2.25–2.35; abdomen without dark spots at the base of tergal bristles; cercal prominence short and almost entirely setulate along median edge (Fig. 7a)	Zaprionus inermis [Cameroon; Central African Republic; Congo; Côte d’Ivoire; Democratic Republic of Congo; Gabon; Kenya; Uganda]
18(16)	BV = 9–11 μm (Fig. 1f); testis short; epandrial phragma with a broad hump at the middle of the anterior margin (Fig. 7c); spermatheca smooth	Zaprionus kolodkinae [Madagascar]
–	BV = 6–8 μm (Fig. 1g); testis long; epandrial phragma with a narrow hump at the dorsal quarter of the anterior margin (Fig. 7d); spermatheca papillate; F1 sometimes with a minute tubercule	Zaprionus verruca [Madagascar]
19(1)	F1 with spines not fused with long bristles at their bases (Figs 2c,d, 8)	20
–	F1 with spines fused with long bristles at their bases (Figs 2e,f)	33
20(19)	F1 with 2 spines pointed in opposite orientation (Fig. 2c)	21
–	F1 with more than 2 spines usually pointed to the same direction (Fig. 2d)	22
21(20)	F1 small (Figs 2c, 8a); abdomen with dark spots at base of bristles	Zaprionus campestris [Cameroon; Côte d’Ivoire; Madagascar, São Tomé and Principé]
–	F1 large (Fig. 8b); abdomen without dark spots at base of bristles	Zaprionus montanus [Burundi; Côte d’Ivoire; Democratic Republic of Congo; Kenya; Rwanda; South Africa]
22(20)	♂ basitarsus without a hairy brush	23
–	♂ basitarsus with a hairy brush	24
23(22)	F1 with 3–4 spines; basalmost spine strong (Fig. 8c)	Zaprionus spinosus [Cameroon; Côte d’Ivoire; Democratic Republic of Congo]
–	F1 with 5 spines internally and sometimes 2 spines externally (Fig. 8d)	Zaprionus spineus [Democratic Republic of Congo]
24(22)	F1 spines differentiated; basalmost spine strong (Figs 2d, 8e)	Zaprionus serratus [Cameroon; Democratic Republic of Congo; Uganda]
–	F1 spines undifferentiated (Fig. 8f–n)	25
25(24)	Wing anterior margin black or darkened (Fig. 9a–c)	26
–	Wing hyaline (Fig. 9d)	29
26(25)	Wing anterior margin black (Fig. 9a,b); F1 spines fine (Fig. 8f,g)	27
–	Wing anterior margin darkened (Fig. 9c); F1 spines robust (Fig. 8h,i)	28
27(26)	F1 with 2–3 spines (Fig. 8g)	Zaprionus fumipennis [Congo; Côte d’Ivoire; Democratic Republic of Congo; Kenya]
–	F1 with 5–6 spines (Fig. 8f)	Zaprionus vrydaghi [Congo; Côte d’Ivoire; Democratic Republic of Congo; Gabon; Tanzania; Uganda]
28(26)	F1 middle bristle borne on a tubercule (Fig. 8h)	Zaprionus tuberarmatus [Cameroon; Democratic Republic of Congo]
–	F1 middle bristle not borne on a tubercule (Fig. 8i)	Zaprionus hoplophorus [Cameroon; Congo]
29(25)	Aedeagal flap absent (Fig. 6c,d)	30
–	Aedeagal flap present	31
30(29)	F1 with a hairy tuft proximally (Fig. 8j); aedeagus short and robust	Zaprionus armatus [Democratic Republic of Congo]
–	F1 without a hairy tuft proximally (Fig. 8k); aedeagus very long and slender	Zaprionus enoplomerus [Cameroon; Côte d’Ivoire]
31(29)	F1 middle bristle borne on a minute tubercule (Fig. 8l); spermatheca voluminous, sclerified at apex and with deep apical introvert (Fig. 6e)	Zaprionus spinipes [Cameroon]
–	F1 middle bristle not borne on a tubercule (Fig. 8m,n); spermatheca not as above	32
32(31)	F1 not broadened, with a series of short bristles (Fig. 8m); spermatheca sclerified (Fig. 6f)	Zaprionus seguyi [Cameroon; Congo; Democratic Republic of Congo]
–	F1 broadened, with a few long bristles (Fig. 8n); spermatheca smooth (Fig. 6g)	Zaprionus spinormatus [Cameroon; Côte d’Ivoire; Nigeria]
33(19)	WV < 15 μm; thorax and abdomen blackish brown	Zaprionus camerounensis [Cameroon; Malawi; Tanzania (loc. n.); Uganda]
–	WV > 15 μm; thorax and abdomen not black	34
34(33)	Abdominal tergal bristles with dark spots basally	35
–	Abdominal tergal bristles without dark spots basally	43
35(34)	Thorax with two incomplete submedian white stripes between two complete dorsocentral stripes (Fig. 1h)	Zaprionus multivittiger [Kenya; Rwanda]
–	Thorax without submedian stripes	36
36(35)	F1 setiferous spines differentiated; basalmost borne on a protruding tubercule (Fig. 2e)	Zaprionus proximus [Kenya; Uganda]
–	F1 setiferous spines undifferentiated	37
37(36)	BV enlarged posteriorly; abdomen dark brown (Fig. 10b,d,e)	38
–	BV not enlarged posteriorly; abdomen light yellow	40
38(37)	Abdomen darker than thorax (Fig. 10b)	Zaprionus koroleu [Benin; Côte d’Ivoire]
–	Abdomen and thorax concolorous (Fig. 10d)	39
39(38)	First and second tarsomeres of the foreleg with strong black spines (Fig. 5c); ♂TL = 1.62–1.68 mm (Fig. 10e); H = 5.2 (Fig. 11d)	Zaprionus lachaisei sp. n. [Tanzania]
–	First and second tarsomeres of the foreleg without strong black spines; ♂TL = 1.44–1.56 mm (Fig. 10d); H = 9.6 (Fig. 11e)	Zaprionus vittiger [Cameroon; Ethiopia; Madagascar; Malawi; South Africa]
40(37)	Head orange tan lighter than thorax (Fig. 10f); hairy brush 1/3 ♂ basitarsus (Fig. 5e); spermatheca without introvert (Fig. 13d)	Zaprionus santomensis sp. n. [São Tomé and Principé]
–	Head and thorax concolorous reddish brown; hairy brush 2/3 ♂ basitarsus; spermatheca with an introvert (Fig. 12)	41
41(40)	♂ aedeagal flap highly serrated apically (Fig. 12a) ; oviscape constricted basally with 8 (rarely 7) peg-like ovisensilla (Fig. 12b); spermatheca length:width = 0.62–0.84 (Fig. 12c)	Zaprionus africanus [Gabon ; Uganda]
–	♂ aedeagal flap highly smooth apically (Fig. 12d,g); oviscape with 6 peg-like ovisensilla (Fig. 12e,h); spermatheca length:width = 0.95–1.16 (Fig. 12f,i)	42
42(41)	♂ aedeagal flap smooth basally (Fig. 12d)	Zaprionus gabonicus [Gabon]
–	♂ aedeagal flap serrated basally (Fig. 12g)	Zaprionus indianus [Argentina; Austria; Benin; Brazil; Cabo Verde; Canary Islands (Spain); Congo; Côte d’Ivoire; Egypt; India; Iran; Israel; Italy; Kenya; Madagascar; Madeira (Portugal); Malawi; Mauritius; Morocco (loc. n.); Mozambique; Niger; Nigeria; Panama; La Réunion (France); São Tomé and Principé; Saudi Arabia; Seychelles; South Africa; Tanzania; United States of America; Uruguay]
43(34)	Abdomen yellow with brown posterior fine stripes on tergites II to IV; TST > 12.0 mm; spermatheca elongated (Fig. 14a)	Zaprionus ornatus [Cameroon; Côte d’Ivoire; Congo; Gabon; Madagascar; South Africa]
–	Abdomen uniformally yellow; TST < 6.0 mm; spermatheca globulous (Figs 14b,c,e)	44
44(43)	TST = 2.6 mm; spermatheca chitinized at base and apex (Fig. 14b); egg with 2 filaments	Zaprionus davidi [Congo; São Tomé and Principé (loc. n.)]
–	TST = 4.0–5.2 mm; egg with 4 filaments	45
45(44)	♂TL = 1.56–1.70 mm; ♂ epandrium not expanded dorsally (Fig. 14d); spermatheca (Fig. 14c)	Zaprionus taronus [Congo (loc. n.); Gabon; Kenya; Malawi; São Tomé and Principé (loc. n.)]
–	♂TL = 1.44–1.50 mm; ♂ epandrium expanded dorsally (Fig. 14e); spermatheca (Fig. 14f)	Zaprionus capensis [South Africa]

## Revised classification of Zaprionus s.s.

Chassagnard and Tsacas (1993) divided Zaprionus s.s. into two groups: inermis and armatus, the latter comprising three subgroups: armatus, tuberculatus and vittiger. The phylogenetic revision of Yassin et al. (2008a) revealed both groups and subgroups to be polyphyletic. However, almost half of the species used in their study lacked DNA sequences, and the discovery and the subsequent molecular analysis of some of these species revealed some new insights (Yassin et al., in press). In light of these findings, a new classification scheme is proposed (Table 2).

Table 2 also shows the breeding niche and the possibility to rear in the laboratory for some species. These two attributes are interrelated, as generalist fruit-breeding species are usually those that can be reared with ease on standard Drosophila medium. Lachaise and Tsacas (1983) reviewed the breeding niche for 12 Zaprionus s.s. species. With the exception of the curious entomophagous ecology of some Afrotropical drosophilids, Zaprionus species share almost all of the known breeding niches of the Afrotorpical fauna, i.e. fruit, flower and decaying tree trunk breeding. Most species are fruit breeders. Some species (e.g., Zaprionus badyi, Zaprionus momorticus, and Zaprionus neglectus) are generalist flower-breeders, whereas two species of the armatus group (Zaprionus fumipennis and Zaprionus vrydaghi) breed exclusively in flowers of Costus afer (Tsacas and Chassagnard 1990). Records of Zaprionus montanus suggest this species to mine bamboo leaves or stems (Graber 1957; Chassagnard 1989). The breeding niche of its sibling species, Zaprionus campestris, is unknown as it was collected by non-selective light or Malaise traps. Zaprionus koroleu was bred from cut palm trunks along with other palm breeding drosophilids of the genera Chymomyza and Scaptodrosophila. However, it appears that no strict association with palm trees has yet evolved in this species as it was able to be reared in the laboratory (although the strain has been lost due to the difficulty of rearing). Other Zaprionus species that were also bred from cut tree trunks included Zaprionus armatus, Zaprionus inermis and Zaprionus ghesquierei.

**Figure 2:**
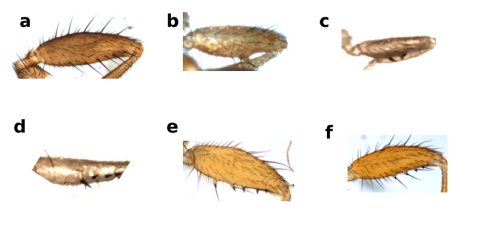
Forefemur of Zaprionus cercus Chassagnard & McEvey, 1992 **a**, Zaprionus mascariensis Tsacas & David, 1975 **b**, Zaprionus campestris Chassagnard, 1989 **c**, Zaprionus serratus Chassagnard, 1989 **d**, Zaprionus proximus Collart, 1937 **e**, and Zaprionus indianus Gupta, 1970 **f**.

**Table 2:** Classification and ecology of the subgenus Zaprionus. Breeding niches are abbreviated as: FL = flowers; FR = fruits; and TR = decaying tree trunk. Ability to be reared in the laboratory (L) is indicated as (+) for species that are reared and (-) for species that are not.

Group	Subgroup	Complex	Species	Authorship	L	Breeding niche	Reference
armatus	armatus	armatus	armatus	Collart, 1937a	-	TR: Ficus sp. (Moraceae)	C37a
						FR: Myrianthus sp. (Cercopiaceae)	TC90
			enoplomerus	Chassagnard, 1989	+	FR: Ficus macrosperma (Moraceae); Ficus sur (Moraceae); Ficus capensis (Moraceae)	C89
			seguyi	Tsacas & Chassagnard, 1990	-	FR	TC90
			spinipes	Tsacas & Chassagnard, 1990	-	?	TC90
			spinoarmatus	Tsacas & Chassagnard, 1990	-	FR: Dacryodes sp. (Burseraceae)	TC90
						TR: Raphia sp. (Arecaceae)	B54
		hoplophorus	hoplophorus	Tsacas & Chassagnard, 1990	-	?
			tuberarmatus	Tsacas & Chassagnard, 1990	-	?
		vrydaghi	fumipennis	Séguy, 1938	-	FL: Costus afer (Costaceae)	TC90
			vrydaghi	Collart, 1937a	-	FL: Costus afer (Costaceae)	B76,C86
	montanus		campestris	Chassagnard, 1989	-	?	C89
			montanus	Collart, 1937b	-	TR: Andropoganeae (Poaceae)	G57
						TR: Bambuseae (Poaceae)	C89
	spinosus		serratus	Chassagnard, 1989	-	FL: Bignoniaceae	C89
			spineus	Tsacas & Chassagnard	-	?	TC90
			spinosus	Collart, 1937a	-	?
inermis			arduus	Collart, 1937b	-	FR: Musa sp. (Musaceae)	C37b
			badyi	Burla, 1954	-	FR	B54
			ghesquierei	Collart, 1937a	-	FR: Citrus sinensis (Rutaceae); Coffea sp. (Rubiaceae); Cola acuminata (Malvaceae); Rollinia sieberi (Annonaceae); Sarcocephalus sp. (Rubiaceae); Psidium sp. (Myrtaceae); Terminalia sp. (Combredaceae); Murraya exotica (Rutaceae); Pseudospondia sp. (Anacridaceae); Myrianthus sp. (Cercopiaceae); Dorstenia sp. (Moraceae); Uapaca sp. (Phyllanthaceae)	C37a
						FR: Musa sp. (Musaceae), Averrhoa carambola (Oxalidaceae); Turraeanthus africana (Meliaceae); Conopharyngia dusissima (Apocynaceae),	B54
						FR: Mangifera indica (Anacardiaceae); Carica papaya (Caricaceae); Persea americana (Lauraceae); Ficus ovata (Moraceae); Musa sp. (Musaceae); Averrhoa carambola (Oxalidaceae); Cyphonandra betacea (Solanaceae); Solanum gilo (Solanaceae)	B76
						FR: Polyalthia sauveolens (Annonaceae); Detarium senegalense (Caesalpiniaceae)	LT83
						FR: Dacryodes sp. (Burseraceae); Hugonia sp. (Linaceae); Parinari sp. (Rosaceae); Gambeya perpulchra (Sapotaceae)	L79
						FR: Cocos romanzoffiana (Palmaceae)	L47
						FR: Pancovia bijuga (Sapindaceae)	L74
						TR: Elaeis guinensis (Palmaceae)	L47
						FR: Ficus thonningii (Moraceae)	C97
			momorticus	Graber, 1957	-	FL: Momordica pterocarpa (syn. M. runsorrica) (Cucurbitaceae)	G57
						FL: Crinum sanderianum (Amaryllidaceae); Crinum jagus (Amaryllidaceae)	L79
						FL: Rothmania whitfieldi (Rubiacea)	L74
			niabu	Burla, 1954	-	FR: Carica papaya (Caricaceae)	B54
	inermis		cercus	Chassagnard & McEvey	+	FR: ex-banana trap	CM92
			inermis	Collart, 1937a	+	FR: Eugenia malaccensis (Myrtaceae)	C37a
						FR: Citrus sp. (Rutaceae); Carica papaya (Caricaceae);	B54
						TR: Raphia sp. (Arecaceae)	B54
						FR: Musa sapientum (Musaceae)	LT83
						TR: Elaeis guineensis (Arecaceae)	LT83
	tuberculatus		kolodkinae	Chassagnard & Tsacas, 1987	+	FR: ex-banana trap	CT87
			mascariensis	Tsacas & David, 1975	+	FR: ex-banana trap	TD75
		sepsoides	sepsoides	Duda, 1939	+	FR: Dacryodes sp. (Burseraceae); Hugonia sp. (Linaceae); Guarea cedrata (Meliaceae); Turraeanthus africanus (Meliaceae); Parinari sp. (Chrysobalanaceae)	L79
						FR: Ficus sur (Moraceae); Ficus lyrata (Moraceae); Ficus macrosperma (Moraceae); Ficus elasticoides (Moraceae); Ficus ovata (Moraceae); Ficus sp. (Moraceae)	L82
						FR: Pandanus candelabrum (Pandanaceae)	R83
						FR: Spondias mombin (Anacardiaceae); Detarium senegalense (Cesalpinaceae); Pentadesma butyraceae (Guttifereae); Treculia africana (Moraceae); Hirtella sp. (Rosaceae); Parinari excelsa (Rosaceae); Nauclea poheguinii (Rubiaceae); Gambeya taiensis (Sapotaceae); Tieghemella heckelii (Sapotaceae)	C86
			tsacasi	Yassin, 2008	+	FR: ex-banana trap	Y08
		tuberculatus	burlai	Yassin, 2008	+	FR: ex-banana trap	Y08
			tuberculatus	Malloch, 1932	+	FR: Santiria trimera (Burseraceae); Dacryodes sp. (Burseraceae); Guarea cedrata (Meliaceae); Parinari sp. (Rosaceae); Parinari excelsa (Rosaceae); Tieghemella heckelii (Sapotaceae)	L79
						FR: Ficus sur (Moraceae); Ficus saussureana (Moraceae); Ficus mucuso (Moraceae); Ficus lutea (Moraceae); Ficus natalensis (Moraceae);	L82, LT83
						FR: Spondias mombin (Anacardiaceae); Detarium senegalense (Cesalpinaceae); Artocarpus sp. (Moraceae); Hirtella sp. (Rosaceae); Uncaria sp. (Rubiaceae); Gambeya taiensis (Sapotaceae)	C86
			verruca	Chassagnard & McEvey, 1992	+	FR: ex-banana trap	CM92
neglectus			neglectus	Collart, 1937	+	FR	B54
						FL: Ipomoea digitata (Convolvulaceae)	B54
						FL: Crinum jagus (Amaryllidaceae); Pentadesma butyracea (Guttiferae); Rothmania whifieldi (Rubiaceae)	C86
						FR: Ficus ovata (Moraceae)	L79
						FR: Treculia africana (Moraceae)	C86
vittiger		davidi	davidi	Chassagnard & Tsacas, 1993	+	FR: ex-banana trap	CT93
			taronus	Chassagnard & Tsacas, 1993	+	FR: Polyalthia sauveolens (Annonaceae); Staudtia gabonensis (Myristicaceae); Cissus dinklagei (Vitaceae)	LT83
		indianus	africanus	Yassin & David, 2008	+	FR: ex-banana trap	Y08b
			gabonicus	Yassin & David, 2008	+	FR: ex-banana trap	Y08b
			indianus	Gupta, 1970	+	FR: ex-banana trap	Y08b
						FR: date palm, guava and citrus	Y09
		ornatus	litos	Chassagnard & McEvey, 1992	-	?	CM92
			ornatus	Séguy, 1933	+	FR: Averrhoa carambola (Oxalidaceae)	B54
						FR: Spondias mombin (Anacardiaceae); Gambeya taiensis (Sapotaceae)	C86
						FR: Ficus sur (Moraceae)	L76
						FR: Ficus macrosperma (Moraceae); Ficus saussureana (Moraceae); Ficus elasticoides (Moraceae); Ficus vogeliana (Moraceae); Ficus mucuso (Moraceae); Ficus ovata (Moraceae); Ficus lutea (Moraceae); Ficus thoningii (Moraceae)	L82, LT83
						FL: Rothmania whitfieldii (Rubiaceae)	L74
		proximus	capensis	Chassagnard & Tsacas, 1993	+	FR: ex-banana trap	CT93
			proximus	Collart, 1937b	+	FR: Cussonia sp. (Araliaceae)	C37b
		sexvittatus	multivittiger	Chassagnard, 1996	+	FR: Rhamnus prinoides (Rhamnaceae)	C96
			sexstriatus	Chassagnard, 1996	+	FR?
			sexvittatus	Collart, 1937c	-	FR: Acokanthera sp. (Apocynaceae)	C37c
						FR: Juniperus procera (Cupressaceae)	C96
		vittiger	camerounensis	Chassagnard & Tsacas, 1993	+	FR: ex-banana trap	CT93
			koroleu	Burla, 1954	+	FR: ex-banana trap
						TR: Raphia sp. (Arecaceae)	B54
			lachaisei	sp. n.	+	FR: ex-banana trap
			santomensis	sp. n.	+	FR: ex-banana trap
			vittiger	Coquillett, 1902	+	FR: ex-banana trap	YP

References: B54: Burla 1954; B76 = Buruga 1976; C37a = Collart 1937a; C37b = Collart 1937b; C86 = Couturier et al. 1986; C89 = Chassagnard 1989; C96 = Chassagnard 1996; C97 = Chassagnard et al. 1997; CM92 = Chassagnard and McEvey 1992; CT87 = Chassagnard and Tsacas 1987; CT93 = Chassagnard and Tsacas 1993; G57 = Graber 1957; L47 = Lepesme 1947; L74 = Lachaise 1974; L76 = Lachaise 1976; L79 = Lachaise 1979; L82 = Lachaise et al. 1982 LT83 = Lachaise and Tsacas 1983; R83 = Rio et al. 1983; TC90 = Tsacas and Chassagnard 1990; TD75 = Tsacas and David, 1975; Y08 = Yassin 2008; Y08b = Yassin et al. 2008b; Y09 = Yassin et al. 2009; YP = Yassin et al., in press.

It is still difficult to estimate with certainty the niches for some of the problematic species in Lachaise and Tsacas’s (1983) review. For example, Zaprionus indianus had almost 80 host plants being the most ecologically diverse drosophilid in the Afrotropical fauna. However, most of the ecological records prior to Tsacas’ (1980) review confused this species with other species of the vittiger group, and even after its identity has been established (Tsacas 1985) the recent discovery of two cryptic species, one of which is also widespread in tropical Africa (Yassin et al. 2008b), sheds doubt on its hosts there. Indeed, Lachaise and Tsacas (1983) described three native host plants from Makokou (Gabon), a locality where the two cryptic species coexist (Yassin et al. 2008b). Although the breeding niches of Zaprionus indianus have been properly determined in its introduced regions in Brazil (Silva et al. 2005; Tidon 2006; Garcia et al. 2008) and the Palearctic region (Yassin et al. 2009), attention has to be paid in the future to determine its breeding niche in its zone of origin. We excluded also the records on the tuberculatus subgroup predating Tsacas et al.’s (1977) discrimination of two sibling species Zaprionus sepsoides and Zaprionus tuberculatus. Records on the Gabonese strain of Zaprionus ornatus in Lachaise and Tsacas (1983) were assigned to Zaprionus taronus since Chassagnard and Tsacas (1993) showed this strain to be misidentified with Zaprionus ornatus by Tsacas (1980).

**Figure 3:**
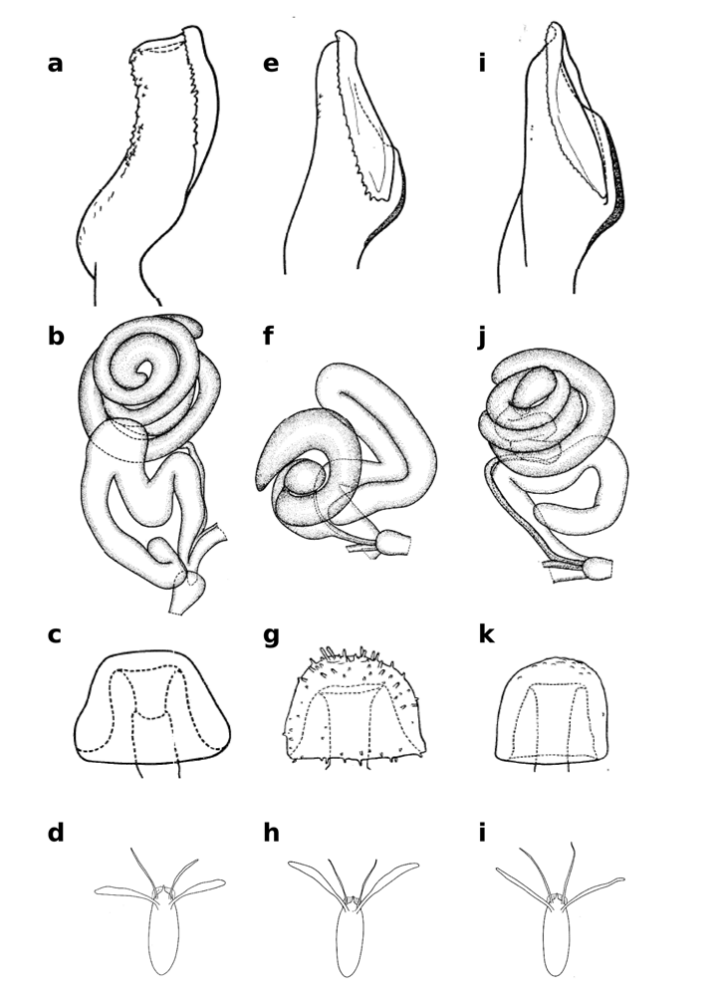
Distiphallus, testis and accessory gland, spermatheca and egg of Zaprionus mascariensis Tsacas & David **a–d**, Zaprionus sepsoides Duda, 1939 **e–h**, and Zaprionus tuberculatus Malloch, 1932 **i–l** [From Tsacas et al. 1977; courtesy of M. T. Chassagnard].

### The armatus group

The armatus group was initially erected to include three subgroups: armatus, tuberculatus and vittiger (Chassagnard and Tsacas 1993). We transferred the tuberculatus subgroup to the inermis group and upgraded the vittiger subgroup to a species group hence restricting the armatus group to the 14 species of the previous armatus subgroup bearing a simple row of spines on F1 (Tsacas and Chassagnard 1990; Fig. 2c,d; Fig. 8). Tsacas and Chassagnard (1990) further subdivided the 14 species of the armatus subgroup to three ‘Ensembles’ I, II and III on the basis of the differentiation of the F1 spines. Yassin et al. (2008a) suggested, using morphological characters of the male genitalia, this subgroup to be polyphyletic. Nonetheless, molecular sequences became later available from a single species, Zaprionus campestris, and its phylogenetic position did not confirm Yassin et al.’s (2008a) placement (Yassin et al., in press). Therefore, Tsacas and Chassagnard’s (1990) subclassification will be retained with slight modifications until new molecular sequences become available. The armatus group is now subdivided into three subgroups: the montanus subgroup with two species bearing two oppositely oriented F1 spines (Ensemble I); the spinosus subgroup with three species bearing a row of differentiated F1 spines (Ensemble II); and the armatus subgroup with nine species bearing a row of undifferentiated F1 spines (Ensemble III). The armatus subgroup is further subdivided into three complexes: the hoplophorus complex with two species bearing differentially oriented strong F1 spines; the armatus complex with five species bearing undifferentially oriented strong F1 spines; and the vrydaghi complex with two species bearing undifferentially oriented fine F1 spines and wings blackened anteriorly.

**Figure 4:**
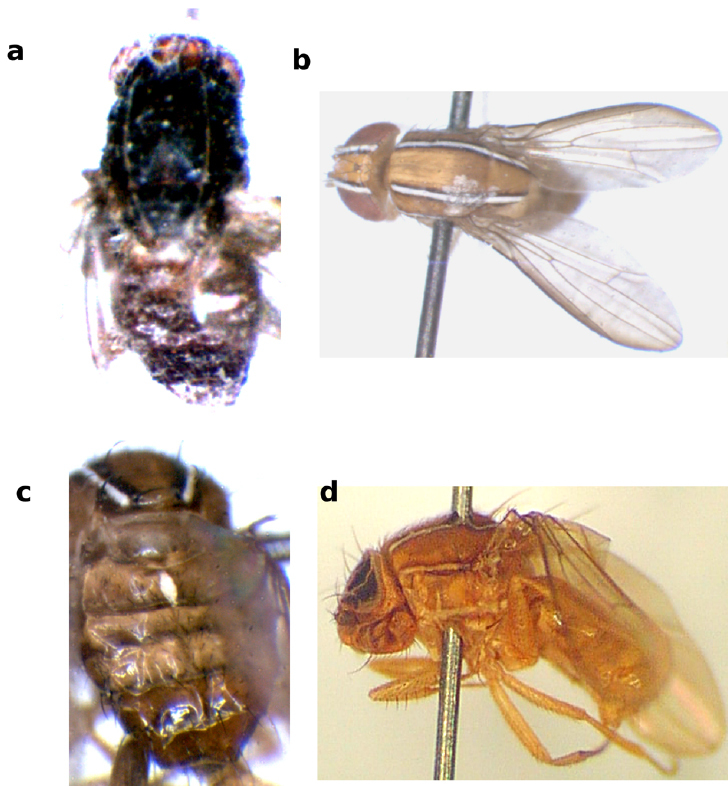
Dorsal views of Zaprionus momorticus Graber, 1957 **a**, Zaprionus badyi Burla, 1954 **b**, abdomen of Zaprionus niabu Burla, 1954 **c**, and lateral view of Zaprionus arduus Collart, 1937 **d**.

### The inermis group

The inermis group comprises species with spineless F1 (Figs 2a,b). The F1 spinelessness is also found in the Oriental subgenus Anaprionus, suggesting a plesiomorphy, and the monophyly of this group was questionable (Chassagnard and Tsacas 1993). Yassin et al. (2008a) suggested on the basis of morphological characters that this group was polyphyletic with two species Zaprionus litos and Zaprionus neglectus being closely related to the armatus and the vittiger groups. These suggestions were confirmed by later molecular analyses (Yassin et al., in press) which also suggested that two other species (Zaprionus sexstriatus and Zaprionus sexvittatus) formed the sister clade with the vittiger group. Four species of the inermis group (Zaprionus arduus, Zaprionus badyi, Zaprionus momorticus and Zaprionus niabu) have not been included in any of these previous studies and their phylogenetic placement remains thus uncertain. Zaprionus ghesquierei forms the earliest branch for the remaining species that are classified here under two subgroups: the inermis subgroup with two species having the short straight aedeagus; and the tuberculatus subgroup with seven species having the curved robust aedeagus. The F1 of several species of tuberculatus subgroup carries a tubercule (Fig. 2b). These two subgroups are closely related to each other as they share the bare and bristleness epandrium (Fig. 7) and the fine serration on the dorsal margin of the aedeagus. These synapomorphies are absent in Zaprionus ghesquierei, Zaprionus arduus, Zaprionus badyi and Zaprionus momorticus. No male specimen has ever been collected for Zaprionus niabu. The tuberculatus subgroup contains two species complexes as suggested by Yassin (2008): the sepsoides complex with two species having short testicules; and the tuberculatus complex with three species having long testicules.

**Figure 5:**
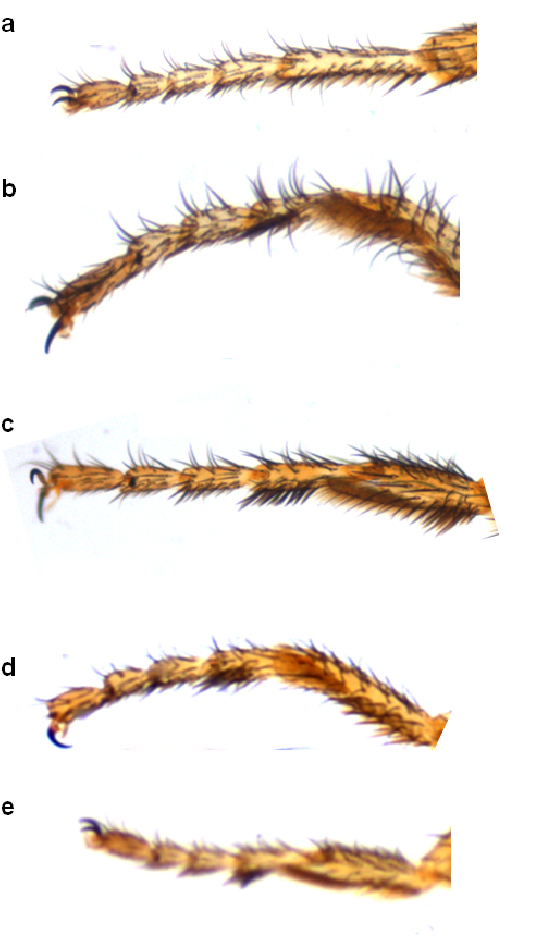
Tarsomeres of male foreleg of Zaprionus neglectus Collart, 1937 **a**, Zaprionus kololdkinae Chassagnard & Tsacas, 1987 **b**, Zaprionus lachaisei Yassin & David, sp. n. **c**, Zaprionus taronus Chassagnard & Tsacas, 1993 **d**, and Zaprionus santomensis Yassin & David, sp. n. **e**.

**Figure 6:**
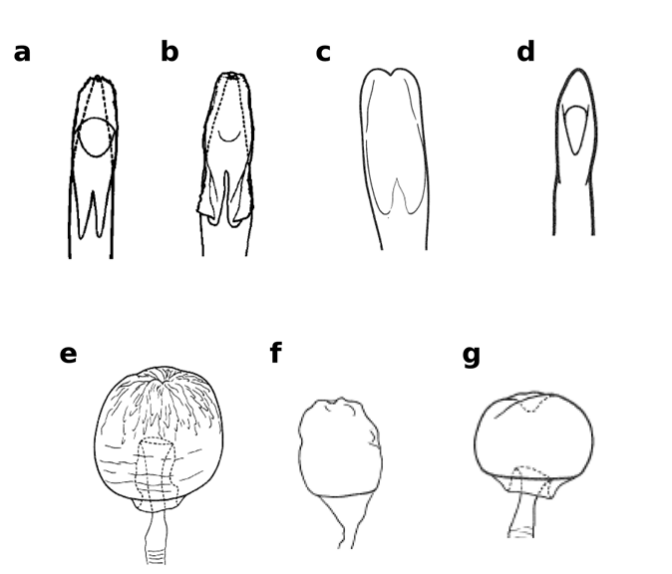
Ventral views of distiphallus of Zaprionus sexvittatus Collart, 1937 **a**, Zaprionus sexstriatus Chassagnard, 1996 **b**, Zaprionus armatus Collart, 1937 **c**, and Zaprionus enoplomerus Chassagnard, 1989 **d**, spermatheca of Zaprionus spinipes Tsacas & Chassagnard, 1990 **e**, Zaprionus seguyi Tsacas & Chassagnard, 1990 **f**, and Zaprionus serratus Chassagnard, 1989 **g**, [From Chassagnard 1989, 1996; Tsacas and Chassagnard 1990; courtesy of M. T. Chassagnard].

### The neglectus group

#### Zaprionus Zaprionus neglectus

Collart

Zaprionus simplex Chassagnard and McEvey 1992, syn. n.

##### Discussion.

Zaprionus neglectus is a continental species lacking F1 ornamentation and the hairy brush on F1 basitarsus in males (Collart 1937b; Fig. 5a). It is the only species previously belonging to the inermis group to lack such a secondary sexual character. Two species of the spinosus subgroup of the armatus group also lack the male hairy brush. Burla (1954) and Lachaise and Tsacas (1983) described that Zaprionus neglectus bred on decaying fruits and in flowers of Ipomoea and Crinum. Chassagnard and McEvey (1992) described a species, Zaprionus simplex, lacking F1 ornamentation and the male hairy brush from Madagascar. They also noted that some specimens were “collected from Crinum sp. flowers but no evidence was found that it bred therein” (p. 322).

We have recently collected a strain of Zaprionus simplex from Crinum sp. in Madagascar and reared it in the laboratory. Burla (1954) noted the presence of two long caecae around the ejaculatory bulb in males of Zaprionus neglectus. Dissection of cultured males of Zaprionus simplex also revealed the presence of long caecae in the Malagasy strain. Wing shape indices were also strongly similar in the original descriptions of the two species. Hence, Zaprionus simplex Chassagnard & McEvey is considered a junior synonym to Zaprionus neglectus Collart. Yassin et al. (2008a) suggested in light of morphological characters Zaprionus simplex, syn. n. to belong to the armatus group, but in the lack of molecular data of any species of this group such relation remains questionable. Indeed, the species has more than 2 epandrial bristles and lacks any F1 ornamentation. Molecular analysis of the Malagasy strain showed the species to be the earliest branch of the subgenus not belonging to any of the three other species groups (Yassin et al., in press). Thus, a group is erected for this single species.

**Figure 7:**
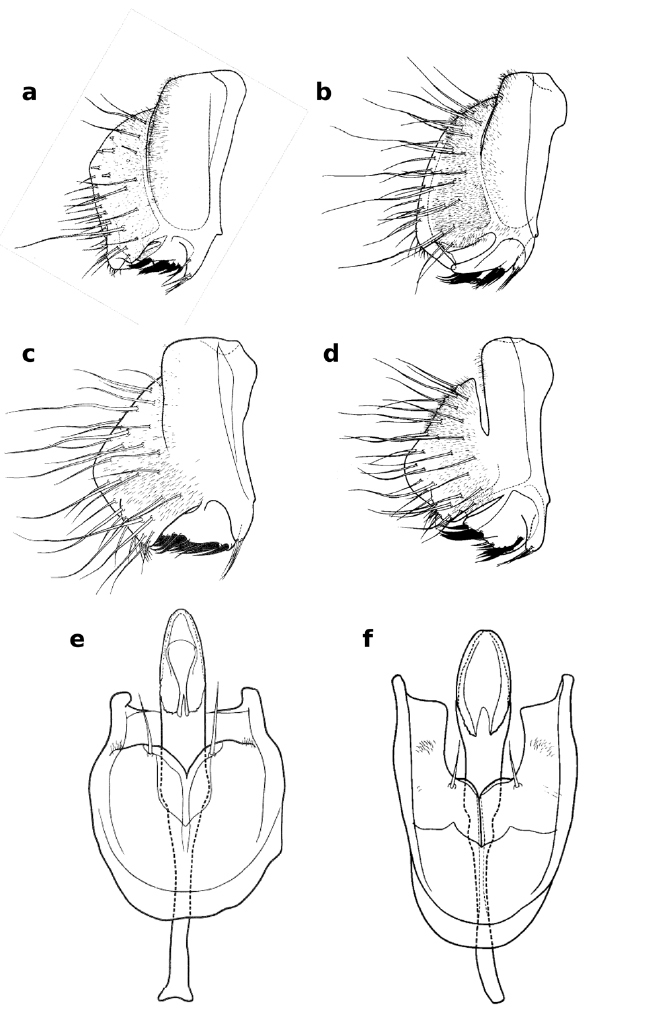
Lateral views of male epandrium and cercus and ventral views of aedeagus and hypandrium of Zaprionus inermis Collart, 1937 **a**, Zaprionus cercus Chassagnard & McEvey, 1992 **b**, Zaprionus kolodkinae Chassagnard & Tsacas, 1987 **c**, **e**, and Zaprionus verruca Chassagnard & McEvey, 1992 **d**, **f** [From Chassagnard and Tsacas 1987; Chassagnard and McEvey 1992; courtesy of M. T. Chassagnard].

**Figure 8:**
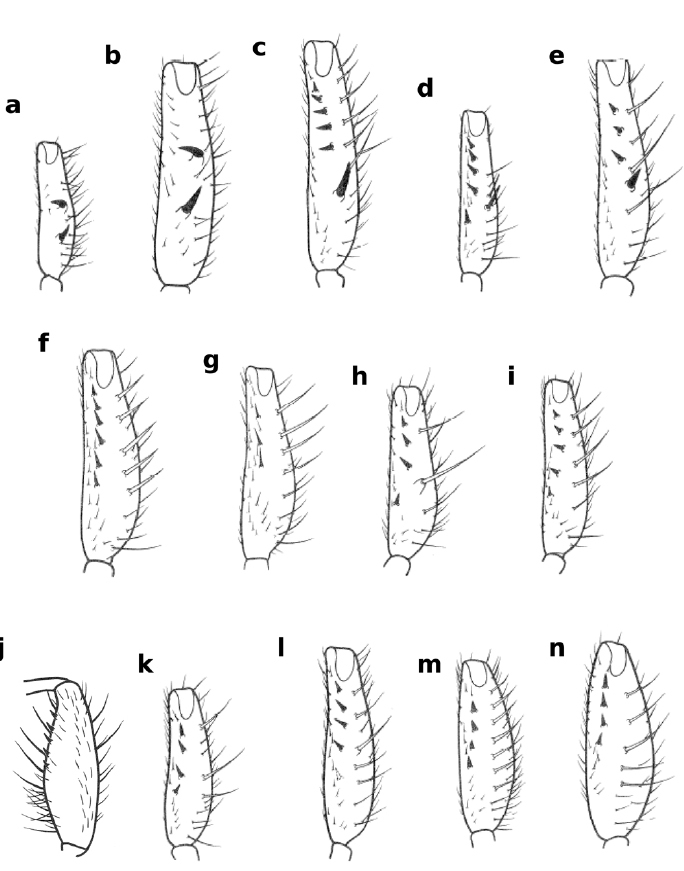
Ventral views of forefemur of Zaprionus campestris Chassagnard, 1989 **a**, Zaprionus montanus Collart, 1937 **b**, Zaprionus spinosus Collart, 1937 **c**, Zaprionus spineus Tsacas & Chassagnard, 1990 **d**, Zaprionus serratus Chassagnard, 1989 **e**, Zaprionus fumipennis Seguy, 1938 **f**, Zaprionus vrydaghi Collart, 1937 **g**, Zaprionus tuberarmatus Tsacas & Chassagnard, 1990 **h**, Zaprionus hoplophorus Tsacas & Chassagnard, 1990 **i**, Zaprionus armatus Collart, 1937 **j**, Zaprionus enoplomerus Chassagnard, 1989 **k**, Zaprionus spinipes Tsacas & Chassagnard, 1990 **l**, Zaprionus seguyi Tsacas & Chassagnard, 1990 **m**, and Zaprionus spinoarmatus Tsacas & Chassagnard, 1990 **n** [From Chassagnard 1989; Tsacas and Chassagnard 1990; courtesy of M. T. Chassagnard].

### The vittiger group

The vittiger group comprises 17 species with usually hairy epandrium carrying more than 2 posterior bristles (Fig. 14d,f). It is mainly characterized by the relatively deep serration of the aedeagal flap. The F1 of most of its species carry composite spines that have bristles fused at their bases and usually are borne on protruding tubercules (Fig. 2e, f). Three species (Zaprionus sexstriatus, Zaprionus sexvittatus and Zaprionus litos) have the unarmed F1 and have been classified in the inermis group (Chassagnard and Tsacas 1993; Chassagnard 1996). Species with F1 bearing composite spines are classified into six complexes: the sexvittatus complex with three species having two additional submedian silvery longitudinal stripes on the thorax (Fig. 1); the ornatus complex with two species having the aedeagal flap weakly serrate apically and smooth basally and greatly extended basally and tapering to a point; the indianus complex with three species having the entirely hairy epandrium and hypandrium and the smooth spermatheca (Fig. 12); the davidi complex with two species having the partially hairy epandrium and rough spermatheca (Fig. 14); the proximus complex with two species having the epandrium enlarged dorsally and tapered ventrally (Fig. 14), the broadened hypandrium and the voluminous cercus lobate at the dorsal margin; and the vittiger complex with five species having the partially hairy epandrium and the smooth spermatheca.

**Figure 9:**
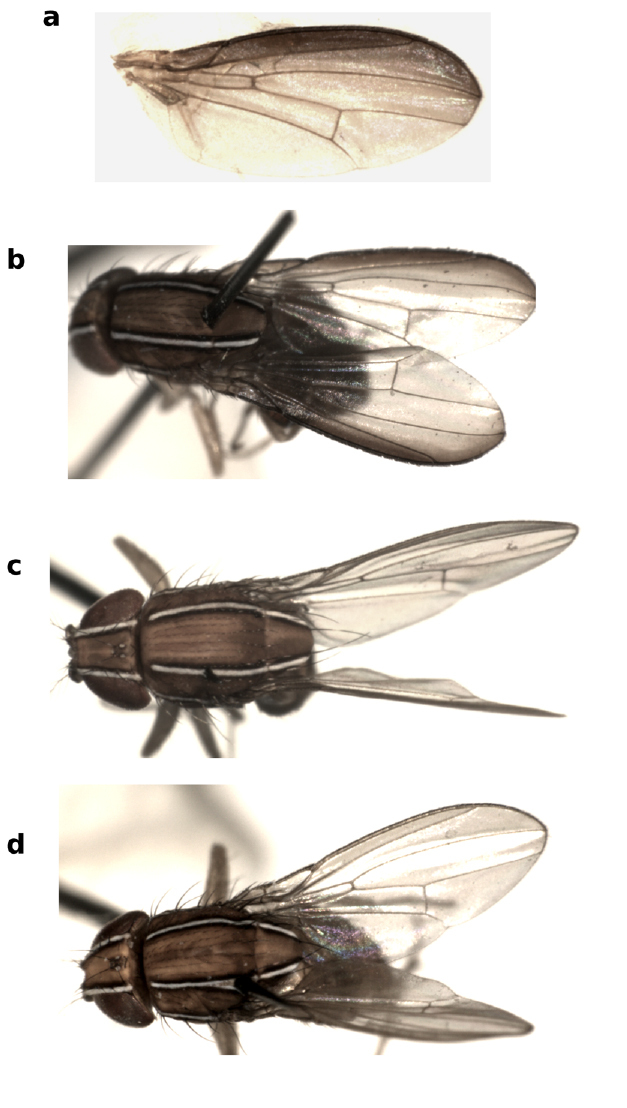
Wing of Zaprionus fumipennis Seguy, 1938 **a**, and dorsal views of Zaprionus vrydaghi Collart, 1937 **b**, Zaprionus hoplophorus Tsacas & Chassagnard, 1990 **c**, and Zaprionus tuberarmatus Tsacas & Chassagnard, 1990 **d**.

**Figure 10:**
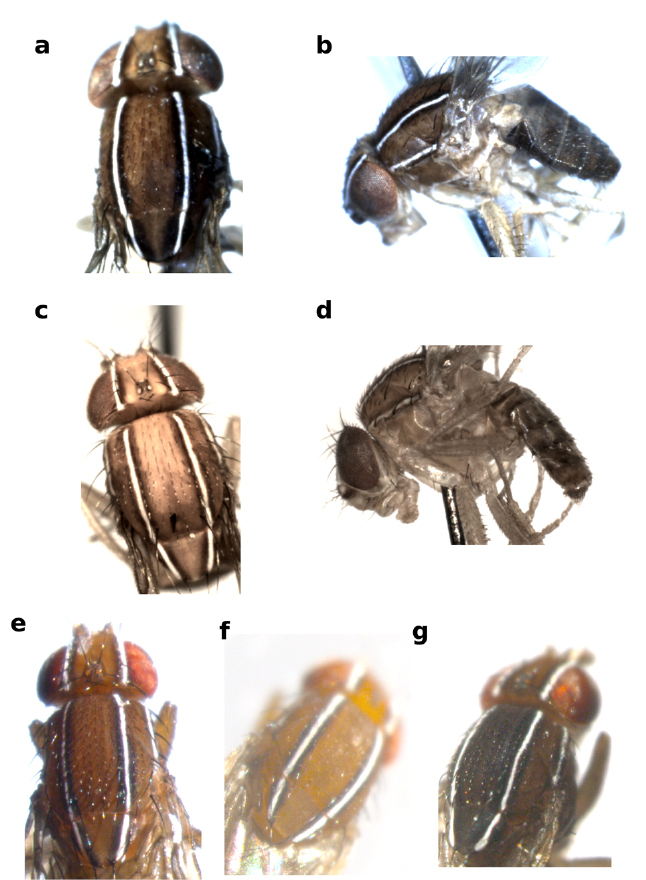
Lateral and dorsal views of Zaprionus koroleu Burla, 1954 **a**, **b**, Zaprionus vittiger Coquillett, 1902 **c**, **d**, Zaprionus lachaisei Yassin & David, sp. n. **e**, Zaprionus santomensis Yassin & David, sp. n. **f**, and Zaprionus camerounensis Chassagnard & Tsacas, 1993 **g**, **h**.

#### Zaprionus Zaprionus ornatus

Séguy

Zaprionus megalorchis Chassagnard and Tsacas 1993, syn. n.

##### Discussion.

Séguy (1933) described a species of the vittiger group from Côte d’Ivoire, which has differentiated F1 composite spines; i.e. the spines are borne on protruding tubercules that decrease in size distally. He called the species Zaprionus ornatus. Collart (1937a) considered this character an intraspecific variation and synonymised Zaprionus ornatus with Zaprionus vittiger. Chassagnard and Tsacas (1993) redescribed Séguy’s female holotype and illustrated the distinctive elongated spermatheca that had also been previously illustrated by Burla (1954) for Zaprionus aff. vittiger. In the same paper, they also described a new species from Congo with the distinctive elongated spermatheca and F1 ornamentation. They called the new species Zaprionus megalorchis and noted that the only difference between it and Zaprionus ornatus was the presence of silver pilosity on the inner side of flagellomere I in Zaprionus ornatus. Yassin et al. (2008a) erected the megalorchis species complex for the two species. However, we have examined a number of strains collected from the type locality of Zaprionus megalorchis and found the flagellomere I pilosity to be polymorphic. We consider thus Zaprionus megalorchis Chassagnard & Tsacas, syn. n. and Zaprionus aff. vittiger Burla, syn. n. to be junior synonyms to Zaprionus ornatus Séguy. Yassin et al. (2008b) have also considered Zaprionus megalorchis (and thus Zaprionus ornatus) a member of the indianus species complex, but it is considered here as belonging to an independent, monophyletic complex along with Zaprionus litos (Yassin et al., in press).

**Figure 11:**
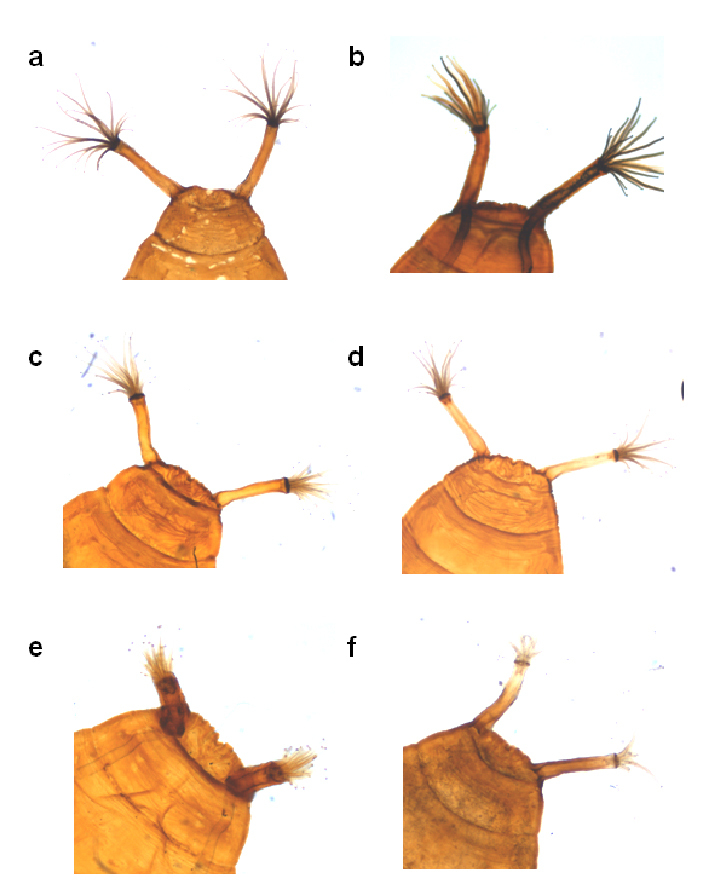
Puparium of Zaprionus neglectus Collart, 1937 **a**, Zaprionus inermis Collart, 1937 **b**, Zaprionus cercus Chassagnard & McEvey, 1992 **c**, Zaprionus santomensis Yassin & David, sp. n. **d**, Zaprionus lachaisei Yassin & David, sp. n. **e** and Zaprionus vittiger Coquillett, 1902 **f**.

#### Zaprionus Zaprionus africanus

Yassin & David

Yassin et al. 2008b 

##### Diagnosis.

**Figure 12:**
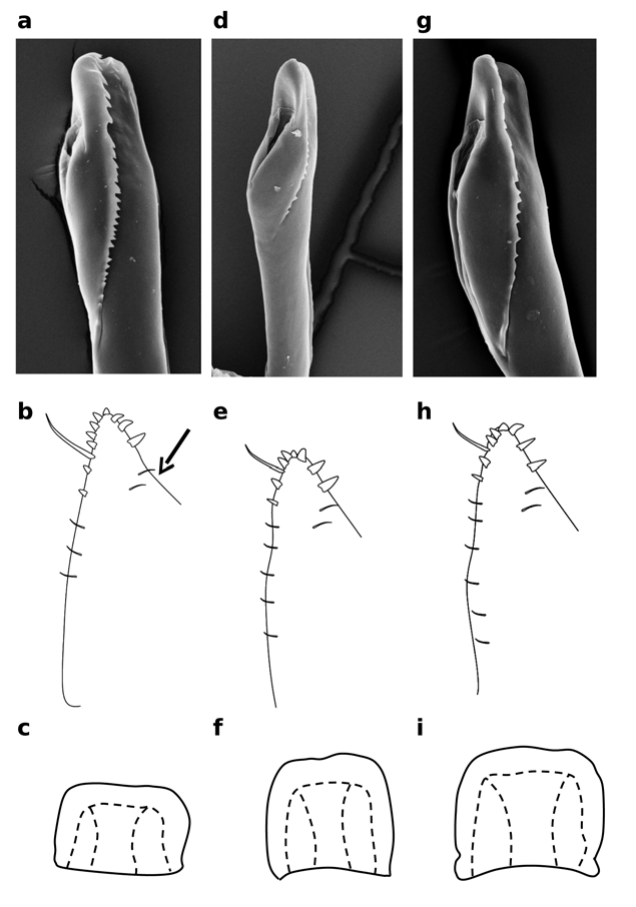
Distiphallus, oviscape and spermatheca of Zaprionus africanus Yassin & David in Yassin et al. 2008b **a-c**, Zaprionus gabonicus Yassin & David in Yassin et al. 2008b **d–f**, and Zaprionus indianus Gupta, 1970 **g–i**.

This species resembles Zaprionus indianus and Zaprionus gabonicus, but can be distinguished from them by the deep serration of the apical margin of the aedeagal flap, the shape of the spermatheca being wider than long and the presence of 8 (rarely 7) peg-like ovisensilla on the oviscape, which is constricted ventrally (Fig. 12).

##### Description.

###### ♂.

TL = 1.38 mm.

###### Head.

Arista with 3 dorsal and 2 ventral rays plus terminal fork; pedicel white, flagellomere I dark brown. Frons orange, without a median stripe but with orbital stripes inwardly bordered with black; ocellar triangle concolorous with frons; hw:fw = 2.42, fw:fl = 0.96. Orbital setae in straight line; or1:or2:or3 = 3:2:3, orbito-index = 1.1, oc:or1 = 1.45, poc:oc = 0.63, iv:ov = 0.88. Face whitish yellow; carina broad and bulbous. Gena broad, o:j = 9.3, o:ch = 6.2. Eye red.

###### Thorax.

Scutum brown, darker than frons, with 2 silvery white stripes. acs in 6 rows in front of adc; adc:pdc = 0.8. Scutellum darker than scutum, with black borders of the stripes expanded posteriorly; bsc:asc = 0.7. Pleura yellow; sterno-index = 0.38. Forefemur with 4–5 spines borne on warts on the anteroventral margin. Male basitarsus with a hairy brush.

###### Wing.

Yellowish. C-index = 2.5, 4v-index = 1.3, 4c-index = 0.9, 5x-index = 1.0, M-index = 0.4, ac-index = 2.5, b/c = 0.7, C3 fringe = 47%, and WL = 2.90 mm.

###### Abdomen.

Entirely yellow with deep dark spots at the bases of tergal setae.

###### Terminalia.

Epandrium densely pubescent throughout its entire length; posterior margin pubescent at dorsal portion with 4 long setae; epandrial ventral lobe with 3 long setae. Surstylus quadrate with two rows of prensisetae. Cercus triangular laterally. Hypandrium densely pubescent at the lateral portion of the paraphyses. Aedeagus expanded apically with a hook-like appendix; aedeagal flap expanded and deeply serrated. Apodeme subequal in length to aedeagus.

###### ♀.

TL = 1.39 mm, resembling male.

###### Terminalia.

Oviscape constricted ventrally, with 8 peg-like and 6 short, marginal setae plus 4 supernumeraries. Spermatheca wide, campaniform and smooth.

###### Egg.

Elliptical with 4, equally long and fine filaments.

###### Larva.

Escaping the culture medium when crowded.

###### Puparium.

Horn-index 9.8.

#### Zaprionus Zaprionus gabonicus

Yassin & David

Yassin et al. 2008b 

##### Diagnosis.

This species resembles Zaprionus indianus, but it can be distinguished from it by the small body size and the total lack of serration on the aedeagal flap (Fig. 12)

##### Description.

###### ♂.

TL = 1.40 mm.

###### Head.

Arista with 3 dorsal and 2 ventral rays plus terminal fork; pedicel white, flagellomere I dark brown. Frons orange, sometimes with highly vestigial median stripe plus orbital stripes inwardly bordered with black; ocellar triangle concolorous with frons; hw:fw = 2.45, fw:fl = 0.85. Orbital setae in straight line; or1:or2:or3 = 1.1:1.0:1.2, orbito-index = 1.1, oc:or1 = 1.4, poc:oc = 0.7, iv:ov = 0.7. Face whitish yellow; carina broad and bulbous. Gena narrow; o:j = 10, o:ch = 4.9. Eye red.

###### Thorax.

Scutum brown, darker than frons, with 2 silvery white stripes. acs in 6 rows in front of adc; adc:pdc = 0.75. Scutellum darker than scutum, with black borders of the stripes expanded posteriorly; bsc:asc = 0.9. Pleura yellow; sterno-index = 0.44. Forefemur with 4–5 spines borne on warts on the anteroventral margin. Male basitarsus with a hairy brush.

###### Wing.

Yellowish. C-index = 2.3, 4v-index = 1.4, 4c-index = 0.8, 5x-index = 1.0, M-index = 0.4, ac-index = 2.2, b/c = 0.6, C3 fringe = 52%, and WL = 2.7 mm.

###### Abdomen.

Entirely yellow with deep dark spots at the bases of tergal setae.

###### Terminalia.

Epandrium densely pubescent throughout its entire length; posterior margin pubescent at dorsal portion with 4 long setae; epandrial ventral lobe with 3 long setae. Surstylus quadrate with two rows of prensisetae. Cercus triangular laterally. Hypandrium densely pubescent at the lateral portion of the paraphyses. Aedeagus slender expanded apically without a hook-like appendix; aedeagal flap expanded and not serrated. Apodeme subequal in length to aedeagus.

###### ♀.

TL = 1.34 mm, resembling male.

###### Terminalia.

Oviscape not constricted ventrally, with 6 (rarely 7) peg-like and 6 short, marginal setae plus 4 supernumeraries. Spermatheca globulous and smooth, not wider than longer.

###### Egg.

Elliptical with 4 equally long and fine filaments.

###### Larva.

Escaping the culture medium when crowded.

###### Puparium.

Horn-index 10.4.

**Figure 13:**
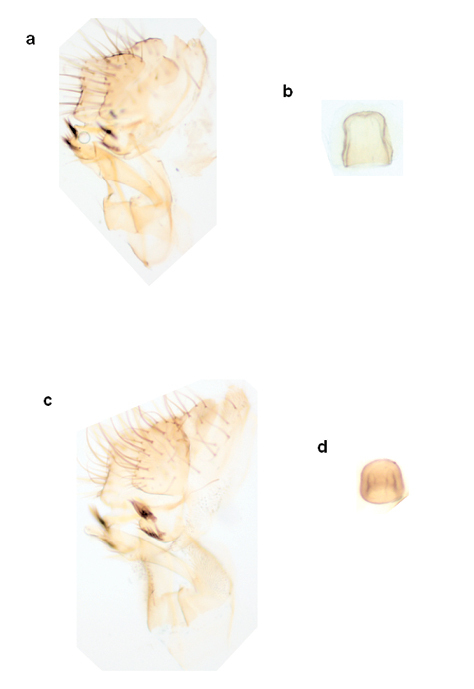
Male genitalia and spermatheca of Zaprionus lachaisei Yassin & David, sp. n. **a**, **b**, and Zaprionus santomensis Yassin & David, sp. n. **c**, **d**.

#### Zaprionus Zaprionus koroleu

Burla

Zaprionus (Zaprionus) beninensis Chassagnard and Tsacas 1993, syn. n.

##### Discussion.

The identity of the dark species Zaprionus koroleu has long been problematic since its description by Burla (1954) from lowland rainforests in Côte d’Ivoire. It had often been confused with another montane dark species in Uganda (Buruga 1976) and Cameroon (Tsacas 1980; Bennet-Clark et al. 1980), which was later described as Zaprionus camerounensis by Chassagnard and Tsacas (1993). Chassagnard and Tsacas (1993) re-examined Burla’s type and considered the enlargement and fusion of BV on the scutellum a characteristic trait of Zaprionus koroleu in the lack of distinctive features of the male genitalia. However, the examination of different strains of Zaprionus vittiger has shown this character to be polymorphic and not exclusive to Zaprionus koroleu. Chassagnard and Tsacas (1993) also noted that Zaprionus koroleu is distinguishable from Zaprionus beninensis in having the thorax and abdomen darker than the frons, whereas in Zaprionus beninensis the abdomen is darker than the frons and the thorax as confirmed by re-examining the type series of Zaprionus beninensis. All species of the vittiger complex are found in high latitudes or altitudes with the exception of Zaprionus koroleu and Zaprionus beninensis. Burla (1954) noted that Zaprionus koroleu was bred in Côte d’Ivoire from decaying Raphia trunk along with other palm breeding drosophilids of the genera Chymomyza and Scaptodrosophila, and this was similar to the breeding niche of Zaprionus beninensis in Benin (fallen trunks of coconut palm; J. R. David, unpublished observations). Both species are, however, generalists as Burla (1954) bred Zaprionus koroleu also from fermenting fruits and as Zaprionus beninensis was maintained in laboratory for almost ten years (Chassagnard and Tsacas 1993). On the basis of these geographical and ecological considerations, only slight differences in pigmentation observed in Zaprionus beninensis and the great morphological similarity of male genitalia, Zaprionus beninensis Chassagnard & Tsacas syn. n. is considered a junior synonym to Zaprionus koroleu Burla.

**Figure 14:**
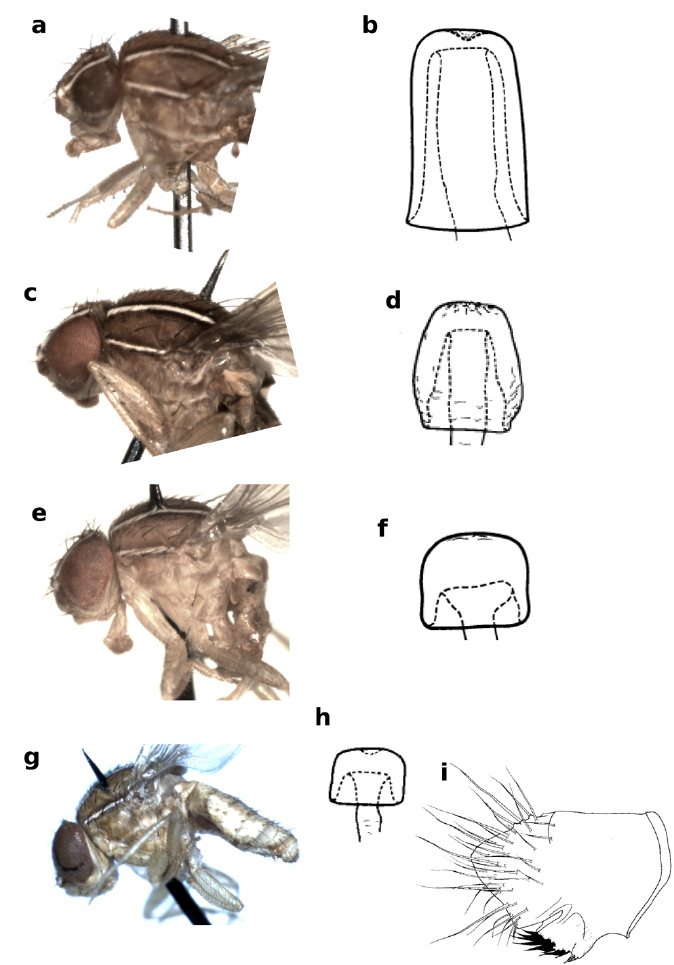
Spermatheca and male epandrium of Zaprionus ornatus Seguy, 1933 **a**, Zaprionus davidi Chassagnard & Tsacas, 1993 **b**, Zaprionus taronus Chassagnard & Tsacas, 1993 **c**, **d**, and Zaprionus capensis Chassagnard & Tsacas, 1993 **e**, **f** [Illustrations from Chassagnard and Tsacas 1993; courtesy of M. T. Chassagnard].

**Figure 15:**
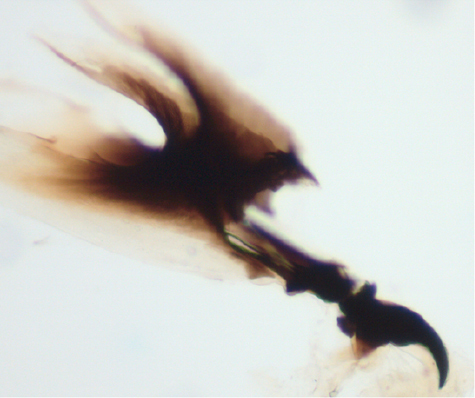
Larval cephalopharyngeal skeleton of Zaprionus sepsoides Duda, 1939.

#### Zaprionus Zaprionus lachaisei

Yassin & David sp. n.

urn:lsid:zoobank.org:act:842BCF21-9ACF-48C1-9B53-9DAC95C49554

##### Diagnosis.

This species resembles Zaprionus vittiger, but has the bigger body size (TL > 1.60 mm), spiniform spines enlarged and blackened on the first two tarsomeres of the foreleg (Fig. 5), and shorter puparial anterior spiracles (H = 5) (Fig. 11). It is also distinguishable by a peculiar behavior of the larvae which do not leave the culture bottle when disturbed or crowded.

##### Description.

###### ♂.

TL = 1.68 mm.

###### Head.

Arista with 3 dorsal and 2 ventral rays plus a terminal fork, pedicel tan. Frons orange-tan with lateral white stripes; median white stripe absent; ocellar triangle raised and darker; hw:fw = 2.04, fw:fl = 1.05. Face pale; carina large; palpus yellow. Gena broad, o:j = 10.2, o:ch = 5.2. Orbital bristles in straight line; or2 very minute, or1:or2:or3 = 7:2:5, orbito-index = 1.4. Ocellar setae long, divergent; oc:or1 = 1.3, poc:oc = 0.5, iv:ov = 0.6. Eye red and densely pilose.

###### Thorax.

Scutum tan, darker than frons, with four white longitudinal stripes continuing on scutellum; white stripes narrow, bordered with large black stripes, especially on the inner side; acs in 6 regular rows anterior to adc and 4 irregular rows between them; psc enlarged, adc:psc = 1.5; adc:pdc = 0.6. Scutellum slightly pointed at the apex, where white spot absent; bsc:asc = 1.3. Sterno-index = 0.6. F1 with 4 setiferous spines not borne on tubercules on the anteroventral margin. Basitarsus of the foreleg with a hairy brush on the ventral margin. Spiniform spines of the first and second tarsomeres of the foreleg enlaged and blackened.

###### Wing.

Dusky; WL:WW = 2.3, C-index = 3.0, 4v-index = 1.5, 4c-index = 0.8, 5x-index = 0.7, M-index = 0.3, ac-index = 2.5, b/c = 0.6, C3 fringe 0.45, WL = 3.8 mm.

###### Abdomen.

Uniformly tan, with dark spots at the bases of tergal bristles.

###### Terminalia (Fig. 13a).

Epandrium densely pubescent at ventral portion; posterior margin pubescent at dorsal portion with 5 long bristles; anterior phragma narrow; epandrial ventral lobe with 3 long bristles. Surstylus quadrate with two rows of prensisetae. Cercus triangular laterally. Hypandrium with a small pubescent patch at the lateral portion of the paraphyses. Aedeagus expanded apically; aedeagal flap expanded and deeply serrate. Apodeme subequal in length to aedeagus.

###### ♀.

TL = 1.76 mm, resembling male.

###### Terminalia.

Oviscape with 8 peg-like and 7 short, marginal setae plus 4 supernumary. Spermatheca large, globulous and smooth (Fig. 13b).

###### Egg.

Elliptical with 4 equally long and fine filaments.

###### Larva.

Not escaping the culture medium when disturbed or crowded.

###### Puparium.

H = 5.0 (Fig. 11d).

##### Distribution

. Tanzania.

##### Type material

. Holotype (male) and allotype (female), Tanzania: East-Usambara Mountains, Amani (870 m), ex type strain ZMI.12, 11-VIII-2008, founder female coll. 25-IX-2002, D. Lachaise. Paratypes: 10 males and 10 females with the same label. Types deposited in MNHN.

##### Discussion

. Attempts to hybridize this strain with others belonging to the vittiger complex have all failed. The species is very prolific and easy to breed in the laboratory.

##### Etymology

. Patronym, in honor of the French Drosophila systematist Dr. Daniel Lachaise (1948–2006), collector of the types of two new species described here.

#### Zaprionus Zaprionus santomensis

Yassin & David sp. n.

urn:lsid:zoobank.org:act:4DE262CC-1AD9-4D00-827B-FC62FC28BACD

Zaprionus sp. B in Araripe et al. 2004

##### Diagnosis.

This species resembles those of the indianus complex in having abdominal tergal spots and F1 spines not borne on protruding tubercule. It can be distinguished from them by the bigger body size, the darker body color mainly in contrast with the frons which is bright orange (Fig. 10f), the wings being dusky rather than hyaline, the smaller hairy brush of the male basitarsus (1/3 of basitarsus) (Fig. 5e), and the lack of an apical introvert in the spermatheca (Fig. 13d).

##### Description.

###### ♂.

 TL = 1.40 mm.

###### Head.

 Arista with 2 dorsal and 3 ventral rays plus terminal fork; pedicel dark brown. Frons orange tan, with vestigial median stripe plus orbital stripes inwardly bordered with black; ocellar triangle blackened; hw:fw = 2.16, fw:fl = 0.8. Orbital setae in straight line; or1:or2:or3 = 3:2:3, orbito-index = 1.8, oc:or1 = 1.5, poc:oc=0.6, iv:ov = 0.4. Face tan. Gena narrow, o:j = 7.6, o:ch = 5.1. Eye red.

###### Thorax.

 Scutum brown, darker than frons, with 2 silvery white stripes. acs in 6 rows in front of adc; adc:pdc = 0.9. Scutellum darker than scutum, with black borders of the stripes expanded posteriorly; bsc:asc = 1.2. Pleura with white pilosity; sterno-index = 0.4. Forefemur with 4 spines not borne on warts on the anteroventral margin. Male basitarsus with a hairy brush.

###### Wing.

 Dusky; WL:WW = 2.3, C-index = 2.8, 4v-index = 1.4, 4c-index = 0.8, 5x-index = 0.9, M-index = 0.3, ac-index = 2.7, b/c = 0.6, C3 fringe 0.40, and WL = 3.2 mm.

###### Abdomen.

 Entirely yellowish, lighter than thorax, with faint dark spots at the bases of tergal setae.

###### Terminalia (Fig. 13c).

Epandrium densely pubescent at ventral portion; posterior margin pubescent at dorsal portion with 3 long setae; anterior phragma slightly humped dorsally; epandrial ventral lobe with 4 long setae. Surstylus quadrate with two rows of prensisetae. Cercus triangular laterally. Hypandrium densely pubescent at the lateral portion of the paraphyses. Aedeagus expanded apically; aedeagal flap expanded and deeply serrate. Apodeme subequal in length to aedeagus.

###### ♀.

 TL = 1.50 mm, resembles male.

###### Terminalia.

 Oviscape with 8 peg-like and 6 short, marginal setae plus 4 supernumeraries. Spermatheca globulous and smooth (Fig. 13d).

###### Egg.

 Elliptical with 4 equally long and fine filaments.

###### Larva.

 Escaping the culture medium when crowded.

###### Puparium.

 Horn-index 10.6.

##### Distribution.

Sao Tomé and Príncipe.

##### Type material.

Holotype (male) and allotype (female), Sao Tomé and Príncipe: Pico de São Tomé Park (1,500 m), ex type strain ZNG, 11-VIII-2008, founder female coll. III-2001, D. Lachaise. Paratypes: 10 males and 10 females with the same label. Types deposited in MNHN.

##### Discussion.

This species resembles Zaprionus proximus, from which it can be distinguished on the basis of F1 ornamentation. An important physiological difference also exists between these species, as Zaprionus santomensis is a very heat-sensitive species since a growth temperature of 25°C is lethal for both sexes and males are sterile at 23 and 24°C (cf. Araripe et al. 2004).

##### Etymology.

The species epithet is in reference to the type locality.

## Comparative anatomy of reproductive system

Many authors described the internal anatomy of some Zaprionus species that can be grown in laboratory (Burla 1954; Throckmorton 1962; Lachaise 1972; Araripe et al. 2004); but with the exception of Tsacas et al.’s (1977) study on the tuberculatus subgroup, little attention has been paid to quantify the differences between the species. Table 3 shows the measurements of some structures in the laboratory strains used in this study. As shown, many measurements give insightful taxonomic differences.

**Table 3:** Comparative morphometry of internal structures of male and female reproductive systems in Zaprionus.

	Male							Female	
	TST	SV	VD	PAR	EC	EB	CAE	SR	SP
Subgenus Anaprionus									
Zaprionus (Anaprionus) bogoriensis	4.4	2.0	0.80	2.6	2.2	0.30	0.6	3.8	0.07
Subgenus Zaprionus									
neglectus group									
Zaprionus (Zaprionus) neglectus	2.8	1.0	0.60	0.7	2.4	0.20	2.0	3.2	0.06
inermis group
Zaprionus (Zaprionus) ghesquierei	1.2	0.6	0.04	2.0	1.1	0.22	1.0	1.5	0.04
Zaprionus (Zaprionus) inermis	1.5	1.1	0.20	2.6	2.1	0.32	0.4	1.0	0.09
Zaprionus (Zaprionus) cercus	1.4	0.9	0.16	2.2	2.0	0.22	1.6	0.9	0.08
Zaprionus (Zaprionus) mascariensis	4.4	0.9	0.40	3.2	1.1	0.22	0.5	7.2	0.12
Zaprionus (Zaprionus) kolodkinae	1.0	0.7	0.20	1.6	2.1	0.20	0.8	0.8	0.06
Zaprionus (Zaprionus) sepsoides	2.0	0.6	0.20	3.2	1.6	0.20	0.1	1.0	0.04
Zaprionus (Zaprionus) tsacasi	1.3	0.8	0.40	3.6	1.2	0.20	0.4	1.2	0.06
Zaprionus (Zaprionus) tuberculatus	3.2	1.2	0.70	2.2	0.9	0.20	0.3	3.6	0.06
Zaprionus (Zaprionus) burlai	4.4	1.0	1.10	2.0	1.3	0.12	0.3	6.3	0.06
Zaprionus (Zaprionus) verruca	3.8	1.6	0.80	2.0	2.0	0.20	1.2	4.0	0.06
vittiger group									
Zaprionus (Zaprionus) ornatus	12.4	7.2	2.20	3.6	0.9	0.30	0.7	12.0	0.18
Zaprionus (Zaprionus) indianus	5.3	2.2	1.30	2.2	1.5	0.30	0.7	4.8	0.16
Zaprionus (Zaprionus) africanus	5.4	1.0	0.70	1.6	1.3	0.30	0.8	3.8	0.07
Zaprionus (Zaprionus) gabonicus	2.5	0.7	0.40	0.7	0.7	0.16	0.4	3.5	0.06
Zaprionus (Zaprionus) davidi	2.6	1.4	0.80	2.0	1.6	0.30	0.6	3.0	0.06
Zaprionus (Zaprionus) taronus	5.2	1.4	1.40	3.2	2.2	0.30	0.8	4.6	0.06
Zaprionus (Zaprionus) capensis	4.0	2.0	0.80	2.6	1.2	0.30	0.6	4.6	0.07
Zaprionus (Zaprionus) proximus	3.6	2.4	2.00	1.4	2.0	0.28	0.3	4.2	0.06
Zaprionus (Zaprionus) santomensis sp. n.	3.6	1.6	1.20	2.0	1.6	0.34	0.7	3.2	0.10
Zaprionus (Zaprionus) lachaisei sp. n.	4.4	2.4	1.30	2.0	2.1	0.30	0.7	4.6	0.10
Zaprionus (Zaprionus) vittiger	4.4	2.4	1.30	2.0	2.4	0.30	0.8	4.2	0.12
Zaprionus (Zaprionus) camerounensis	4.2	2.0	0.70	3.2	1.2	0.20	0.6	4.5	0.09

TST = testis; SV = seminal vesicle; VD = vas deferens; PAR = paragonia (accessory gland); EC = ejaculatory bulb; CAE = caecum; SR = seminal receptacle; SP = spermatheca.

**Table 4:** Measurements of immature stages in Zaprionus species grown under the same laboratory conditions.

	Egg		Puparium	
	EL:El	PF:EL	PL:Pl	H
Subgenus Anaprionus				
Zaprionus (Anaprionus) bogoriensis	3.45	1.13	2.54	9.3
Subgenus Zaprionus				
neglectus group				
Zaprionus (Zaprionus) neglectus	2.90	0.83	2.31	15.3
inermis group				
Zaprionus (Zaprionus) ghesquierei	3.00	0.54	2.54	9.4
Zaprionus (Zaprionus) inermis	3.26	1.13	2.62	13.1
Zaprionus (Zaprionus) cercus	2.90	0.97	2.40	10.3
Zaprionus (Zaprionus) mascariensis	2.91	0.73	2.47	6.8
Zaprionus (Zaprionus) kolodkinae	2.75	0.97	2.43	9.0
Zaprionus (Zaprionus) sepsoides	3.10	0.90	2.57	8.6
Zaprionus (Zaprionus) tsacasi	2.73	0.90	2.53	8.4
Zaprionus (Zaprionus) tuberculatus	2.86	0.90	2.59	7.0
Zaprionus (Zaprionus) burlai	3.00	0.91	2.29	7.2
Zaprionus (Zaprionus) verruca	3.40	0.88	2.31	10.6
vittiger group				
Zaprionus (Zaprionus) ornatus	3.18	1.14	2.52	10.0
Zaprionus (Zaprionus) indianus	3.44	0.81	2.49	8.3
Zaprionus (Zaprionus) africanus	3.26	0.90	2.46	9.8
Zaprionus (Zaprionus) gabonicus	3.33	0.83	2.43	10.4
Zaprionus (Zaprionus) davidi	3.05	1.16	2.54	10.5
Zaprionus (Zaprionus) taronus	2.87	0.91	2.29	12.0
Zaprionus (Zaprionus) capensis	2.43	1.00	2.45	9.8
Zaprionus (Zaprionus) proximus	3.67	1.06	2.44	10.6
Zaprionus (Zaprionus) santomensis sp. n.	2.86	0.60	2.24	10.6
Zaprionus (Zaprionus) lachaisei sp. n.	3.28	0.78	2.64	5.0
Zaprionus (Zaprionus) vittiger	3.20	1.06	2.65	9.3
Zaprionus (Zaprionus) camerounensis	3.00	0.93	2.56	11.0

EL = egg length; El = egg width; PL = puparium length; Pl = puparium width; H = horn-index.

### Male reproductive system

Testis length (TST) ranges from 1.0 mm in Zaprionus kolodkinae to 12.4 mm in Zaprionus ornatus. The Oriental species, Zaprionus (Anaprionus) bogoriensis, has TST of 4.4 mm which approaches that of the mean of the African species (3.7 ± 0.5 mm). Species of the inermis group can be classidfied under two categories: those with small testis ranging from 1.0 to 2.0 mm (Zaprionus inermis, Zaprionus cercus, Zaprionus kolodkinae, Zaprionus sepsoides and Zaprionus tsacasi), and those with large testis ranging from 3.2 to 4.4 mm (Zaprionus mascariensis, Zaprionus tuberculatus, Zaprionus burlai and Zaprionus verruca). Species of the last category are all members of the tuberculatus subgroup which also include some species of the first category, and TST presents a very informative taxonomic clue (Fig. 3; Tsacas et al. 1977; Yassin 2008). In the vittiger group, Zaprionus ornatus with its very long testis (TST = 12.4 mm) is particular. The remaining species can be classified under four discontinuous categories: Zaprionus gabonicus and Zaprionus davidi with TST from 2.5 to 2.6 mm; Zaprionus proximus and Zaprionus santomensis sp. n. with TST of 3.6 mm; Zaprionus capensis, Zaprionus camerounensis, Zaprionus vittiger and Zaprionus lachaisei sp. n. with TST from 4.0 to 4.4 mm; and Zaprionus indianus, Zaprionus africanus and Zaprionus taronus with TST from 5.2 to 5.4 mm. Unlike in the inermis group, the categories of the vittiger group do not reflect any phylogenetic trend.

The seminal vesicle (SV) is the part of the vas deferens that has undergone a differentiation for sperm storage. It ranges from 0.6 mm in Zaprionus ghesquierei and Zaprionus sepsoides to 7.2 mm in Zaprionus ornatus, with the mean of 1.6 ± 0.3 mm in African Zaprionus. Species of the inermis group tend to have small SV, ranging from 0.6 to 1.6 mm, whereas species of the vittiger group have larger SV, ranging from 0.7 to 2.4 mm (excluding Zaprionus ornatus).

The vas deferens (VD) ranges from 0.04 mm in Zaprionus ghesquierei to 2.20 mm in Zaprionus ornatus. The quasi-absence of VD in Zaprionus ghesquierei is exceptional as the next value to it is 0.20 mm in a number of species of the inermis group (Zaprionus inermis, Zaprionus kolodkinae and Zaprionus sepsoides). Indeed, Throckmorton (1962) described VD morphology in a laboratory strain of Zaprionus ghesquierei. The 12 males he dissected “were variable, showing two major types with only slight integradation between them” (pp. 232). The VDs of three males were quasi-absent like the one described here, whereas those of the remaining nine males were “somewhat longer and associates closely with the ventral surface of the paragonia.” We did not find this polymorphism in the few individuals dissected. The longest VD in the inermis group is found in Zaprionus burlai (VD = 1.1 mm), and it is greater than VDs of its two relatives (0.7 mm in Zaprionus tuberculatus and 0.8 mm in Zaprionus verruca).

The ejaculatory bulb of Zaprionus species is moderately large, rounded and bearing long posterior caecae (Throckmorton 1962). In the vittiger species group, the posterior caecae are branched several times, whereas in the remaining African and Oriental species the caecae are unbranched. The length of the caecae (CAE) ranges from 0.1 mm in Zaprionus sepsoides to 2.0 mm in Zaprionus neglectus. The long CAE of Zaprionus neglectus is exceptional (Burla 1954) and it was used as one of the arguments to synonymize Zaprionus neglectus Burla with Zaprionus simplex Chassagnard & McEvey. CAE can also be used to distinguish Zaprionus cercus (CAE = 1.6 mm) from its sibling species Zaprionus inermis (CAE = 0.4 mm), which has particularly small CAE. Lachaise (1972) also noted that CAE of Zaprionus inermis was about 0.6 mm. Zaprionus verruca has exceptional long CAE of 1.2 mm in the tuberculatus subgroup, that can easily distinguish it from its two sibling species Zaprionus tuberculatus and Zaprionus burlai (CAE = 0.3 mm).

### Female reproductive system

The seminal receptacle (SR) ranges from 0.8 mm in Zaprionus kolodkinae to 12.0 mm in Zaprionus ornatus. As with TST, species of the vittiger group tend to have larger SR than those of the inermis group. The correlation between TST and SR is a well-established fact in the Drosophilidae, although the correlation is thought to be functional rather than genetic (Joly and Bressac 1994). This correlation is obvious in Zaprionus (r = 0.93; P < 0.001). SR can distinguish Zaprionus burlai females (SR = 6.3 mm) from Zaprionus tuberculatus (SR = 3.6 mm), and Zaprionus indianus (SR = 4.8 mm) from Zaprionus africanus (SR = 3.8 mm) and Zaprionus gabonicus (SR = 3.5 mm).

Burla (1954) provided the first account of the morphology of the spermatheca (SPR) in Zaprionus species from Côte d’Ivoire, and illustrations of spermathecae became a taxonomic routine in all descriptions following his study (Figs 3, 6, 12, 13). The elongate form of the spermatheca of Zaprionus ornatus is characteristic and it was one of the arguments for considering Zaprionus megalorchis Chassagnard and Tsacas syn. n. and Zaprionus aff. vittiger Burla as junior synonyms for this species (Fig. 13). We dissected 10 females per species in the indianus complex and found that in Zaprionus africanus the width of the spermatheca was always relatively greater than its length, whereas in its two cryptic species Zaprionus indianus and Zaprionus gabonicus, the spermatheca length and width were subequal (Fig. 12). In the tuberculatus species subgroup, it is the shape rather than the length of the spermatheca which provides the best taxonomic clues (Fig. 3).

## Immature stages

### Egg

The eggs of species of the Oriental subgenus Anaprionus have two filaments (Bock 1966; Bock and Baimai 1967), whereas in African Zaprionus s.s. they have four filaments. A single exception in Zaprionus s.s. is Zaprionus davidi whose eggs have also two filaments (Chassagnard and Tsacas 1993). However, they still can be distinguished from those of the Oriental species by the presence in the latter of a thin, chitinized crest at the apex of the operculum.

The length of the filaments varies between species (Table 3). In Zaprionus momorticus, the four filaments are very short (Graber 1957). In most species, however, the posterior (dorsal) filaments are usually longer than the anterior (ventral) ones. In some species (Zaprionus mascariensis, Zaprionus kolodkinae, Zaprionus sepsoides and Zaprionus tsacasi) of the Zaprionus tuberculatus species subgroup (Fig. 3), the posterior filaments are usually elongated and spatulate near the apex.

### Larva

Larvae of the genus Zaprionus are all of the amphipneustic type as in other drosophilid flies (Okada 1968). In all instars of both subgenera, the larval cephalopharyngeal skeleton is smooth lacking any dentition (Fig. 15). In all species, when cultures are crowded, the mature larvae climb up the bottle and often escape through the plug, and die from desiccation (Bock 1966; David et al. 2006). Zaprionus lachaisei sp. n. is the only species of which larvae do not show this peculiar behavior, and this makes its laboratory culture an easier.

### Puparium

Puparia of the two subgenera are reddish brown in color (Fig. 11). The puparial length (PL) ranges from 2.82 mm in Zaprionus gabonicus to 4.58 mm in Zaprionus inermis, in complete concordance with the differences of body size in the adults (Yassin and David, in press). The only other species with PL exceeding 4.00 mm are Zaprionus lachaisei sp. n. (PL = 4.30 mm) and Zaprionus bogoriensis (PL = 4.20 mm). The puparial shape (PL:Pl) ranges from 2.24 in Zaprionus santomensis sp. n. to 2.65 in Zaprionus vittiger. Interestingly this ratio can serve in discriminating puparia of some close species such as between: Zaprionus inermis (2.62) and Zaprionus cercus (2.40), and Zaprionus tuberculatus (2.59) and Zaprionus burlai (2.29).

The horn-index (H) is a classical taxonomic measurement in drosophilid systematics. H ranges from 5.0 in Zaprionus lachaisei sp. n. (Fig. 11d) to 15.3 in Zaprionus neglectus (Fig. 11A) with the mean of 9.7 ± 0.4 in African Zaprionus (9.3 in the Oriental species Zaprionus bogoriensis). With the exception of the two extremes, H ranges from 6.8 to 13.1. In the tuberculatus species complex, H discriminates Zaprionus verruca (H = 10.6) from its two sibling species, Zaprionus tuberculatus (H = 7.0) and Zaprionus burlai (H = 7.2).

Another important taxonomic character of the puparium is the branches of the anterior spiracle. In all Zaprionus species, these branches are of the clubbed type (Okada 1968). The arrangement of the branches on the stalk is of the type Y in which pseudocentral branches (sensu Okada 1968) are absent. The number of branches tends to vary from 11 to 14 in the inermis species group, and from 15 to 17 in the vittiger group. A particular exception is found in Zaprionus inermis where the number of branches ranges from 18 to 21 (Fig. 11b). This facilitates the discrimination of its puparia from those of its sibling species, Zaprionus cercus, which has 11 to 13 branches (Fig. 11c).

## Supplementary Material

XML Treatment for Zaprionus Zaprionus neglectus

XML Treatment for Zaprionus Zaprionus ornatus

XML Treatment for Zaprionus Zaprionus africanus

XML Treatment for Zaprionus Zaprionus gabonicus

XML Treatment for Zaprionus Zaprionus koroleu

XML Treatment for Zaprionus Zaprionus lachaisei

XML Treatment for Zaprionus Zaprionus santomensis
